# Proteomic profiling of iPSC and tissue-derived MSC secretomes reveal a global signature of inflammatory licensing

**DOI:** 10.1038/s41536-024-00382-y

**Published:** 2025-02-04

**Authors:** Margeaux Hodgson-Garms, Matthew J. Moore, Mikaël M. Martino, Kilian Kelly, Jessica E. Frith

**Affiliations:** 1 Department of Materials Science and Engineering, Monash University, Melbourne, VIC Australia; 2 Cynata Therapeutics, Melbourne, VIC Australia; 3 Australian Regenerative Medicine Institute, Melbourne, VIC Australia; 4 Victorian Heart Institute, Monash University, Melbourne, VIC Australia

**Keywords:** Extracellular signalling molecules, Proteomics, Mesenchymal stem cells, Reprogramming, Stem-cell research

## Abstract

Much of the therapeutic potential of mesenchymal stromal cells (MSCs) is underpinned by their secretome which varies significantly with source, donor and microenvironmental cues. Understanding these differences is essential to define the mechanisms of MSC-based tissue repair and optimise cell therapies. This study analysed the secretomes of bone-marrow (BM.MSCs), umbilical-cord (UC.MSCs), adipose-tissue (AT.MSCs) and clinical/commercial-grade induced pluripotent stem cell-derived MSCs (iMSCs), under resting and inflammatory licenced conditions. iMSCs recapitulated the inflammatory licensing process, validating their comparability to tissue-derived MSCs. Overall, resting secretomes were defined by extracellular matrix (ECM) and pro-regenerative proteins, while licensed secretomes were enriched in chemotactic and immunomodulatory proteins. iMSC and UC.MSC secretomes contained proteins indicating proliferative potential and telomere maintenance, whereas adult tissue-derived secretomes contained fibrotic and ECM-related proteins. The data and findings from this study will inform the optimum MSC source for particular applications and underpin further development of MSC therapies.

## Introduction

Mesenchymal stromal cells (MSCs) are a heterogeneous population of multipotent cells that can be isolated from virtually all organs and tissues^1^ or generated from induced pluripotent stem cells (iMSCs)^2^. They are broadly defined by the International Society for Cell and Gene Therapy (ISCT) as plastic adherent stromal cells; expressing CD73, CD90 and CD105 surface antigens in the absence of CD34, CD45, CD14, CD19, and HLA-DR; and with the capacity to differentiate along osteogenic, chondrogenic, and adipogenic linages in vitro^3^. Beyond this, MSCs also possess significant immunomodulatory capacity and have been widely documented to aid tissue regeneration and repair^4,5^. This has led to MSCs becoming one of the most extensively studied cell therapies worldwide. To fully realise their clinical potential, however, fundamental aspects of MSC biology and how exactly they exert their immunomodulatory and pro-regenerative functions, must be clarified. This is complicated by the dynamic nature of MSCs and the heterogeneity they exhibit between tissue sources, donors, and within individual populations^6^.

The range of clinical indications to which MSCs are applied is incredibly diverse, but typically targets two pathophysiological categories: immune and inflammatory conditions, such as graft-versus-host disease^7^ and Crohn’s disease; or tissue repair and regeneration applications, including soft tissue wound healing^8^ and cardiac repair^9^. The breadth of these applications is driven by the fact that MSCs exhibit several distinct phenotypes, between which they switch in response to microenvironmental cues, a quality referred to as plasticity^10,11^. The current model of MSC plasticity, describes three unique phenotypes; resting MSCs, which are thought to be responsible for maintaining the haematopoietic niche and stromal tissues; a pro-inflammatory phenotype, referred to as MSC1; and, an immuno-suppressive phenotype, referred to as MSC2^12–16^. This nomenclature mirrors the system applied to the M1/M2 polarisation of macrophages, reflecting similar engagement throughout the wound healing process. This model of MSC plasticity suggests early tissue-injury signals result in pro-inflammatory MSC1 licensing, causing MSCs to home to the site of an injury, recruit peripheral immune cells and participate in the early phases of wound healing. Subsequent anti-inflammatory licensing to an MSC2 phenotype successively aids in resolution of the immune response and wound healing process^17–19^.

Whilst MSCs were originally predicted to repair injured tissues through engraftment and subsequent differentiation^20–22^, most studies in which MSCs have been administered lack evidence of long-term cell survival, despite showing functional improvements^22–25^. Indeed, up to 95% of systemically administered MSCs are cleared from the bloodstream in less than 5 min^26–28^ and repair of injured tissues is often observed too rapidly to be explained by direct differentiation of administered MSCs^29^. As such, the therapeutic potential of MSCs is now strongly attributed to their secretion of bioactive and paracrine factors, which are known to change rapidly, reflecting the phenotypic plasticity of MSCs^23,30–37^.

The MSC secretome consists of a complex milieu of biologically active factors, including extracellular vesicles, chemokines and receptors (CCL2, CCL5, CXCL8-11), mitogenic and angiogenic growth factors (FGF, VEGF, TGF-β), inflammatory cytokines (IL-6, IL-8), immunomodulatory factors (indoleamine 2,3-dioxygenase (IDO), MHC-I, MCH-II) and extracellular matrix (ECM) components (collagens, fibronectins, integrins)^38–42^. While the roles that individual factors play in driving the clinically relevant functions of the MSC secretome are regularly subject to review^32,43,44^, there remains little consensus on the complete MSC secretory profile, suggesting that a more comprehensive understanding is still required to effectively capitalise on their therapeutic potential.

Of the three established MSC phenotypes, resting and MSC2 are of the greatest clinical interest as it is these phenotypes that likely drive the potential of MSCs as pro-regenerative and immunosuppressive agents^15,16^. Despite this, inflammatory licensing is often overlooked, with comparative and functional studies often focusing solely on resting MSCs. As a result, little is known of how the secretome changes with licensed phenotypes. This is of particular importance as many of the targeted applications of MSCs involve delivery of live and responsive cells into a dynamic in vivo microenvironment.

Importantly, as with other aspects of MSC identity, MSC secretory profiles also differ between donors and between tissue sources^32,45^ and there is a lack of consensus as to how the tissue specific identity of MSCs affects their inflammatory response and plasticity. This constitutes a significant gap in our understanding that impacts the ability to effectively select optimal MSC lines for specific applications. Specifically, the secretome of iMSCs has yet to be fully characterised under either resting or inflammatory licensed conditions and there is currently no information available as to if, or how, iMSCs respond to inflammatory microenvironments.

This study addressed these questions by using high resolution two-dimensional liquid chromatography-tandem mass spectrometry (LC-MS/MS) to perform a systematic, cross-comparison of the secretomes of MSCs from multiple tissue sources and iPSC-backgrounds, both in resting and licensed states. To address the heterogeneity that exists between MSC tissue-sources and donors, as well as the lack of information on the secretome of iMSCs, this study profiled multiple batches of clinical-grade iMSCs alongside an additional commercial-grade iMSC line, in parallel with multiple tissue-derived MSC populations, sourced from triplicate donors of bone marrow, adipose tissue, and umbilical cord. This has generated a comprehensive atlas of the MSC secretome, allowing identification of a conserved profile of MSC2 inflammatory licensing and a robust comparison of resting and MSC2 secretomes. Furthermore, the data allows the comparison of iPSC and tissue-derived MSC secretomes and provides novel evidence that iMSCs are capable of comparable inflammatory licensing.

From this dataset, comparisons may also be drawn between the secretomes of either resting or licensed MSCs derived from tissue-specific MSC sources; between adult and natal tissue sources; between clinical and commercial-grade iMSCs; and between individual donors or batches of each MSC population. This data set serves as a robust framework from which to make preliminary predictions towards the therapeutic potential of different MSCs and identify the MSC source and phenotype most likely to have optimal therapeutic effects for each of the varying target applications.

## Results

To compare the immune plasticity and secretory profiles of iPSC and tissue-derived MSCs a panel of 13 MSC lines was established. This consisted of three donors each of bone marrow (BM.MSCs 1-3), adipose tissue (AT.MSCs 1-3) and umbilical cord (UC.MSCs 1-3)-derived MSCs, three batches of Cynata Therapeutics’ CYP001^TM^ clinical iMSCs (CYN.iMSCs 1-3) and one batch of commercially available iMSCs (Cellular Dynamics Incorporated) (CDI.iMSC 4). This panel allowed comparisons between iPSC and tissue-derived MSCs, between MSCs from different tissue sources (including both adult and natal tissues), and between MSCs from different batches/donors of the same source (Supplementary Table 1). The basic characteristics of the MSC lines were assessed to confirm compliance with the ISCT minimal criteria. All MSC lines successfully adhered to, and proliferated on, tissue-culture plastic. They expressed CD73, CD90, and CD105 surface markers in the absence of CD14, CD19, CD34, CD45, and HLA-DR (Supplementary Fig. 1) and demonstrated the ability to differentiate in vitro along the osteogenic, adipogenic, and chondrogenic lineages (Supplementary Fig. 2).

### Inflammatory licencing of MSCs

**Fig. 1:**
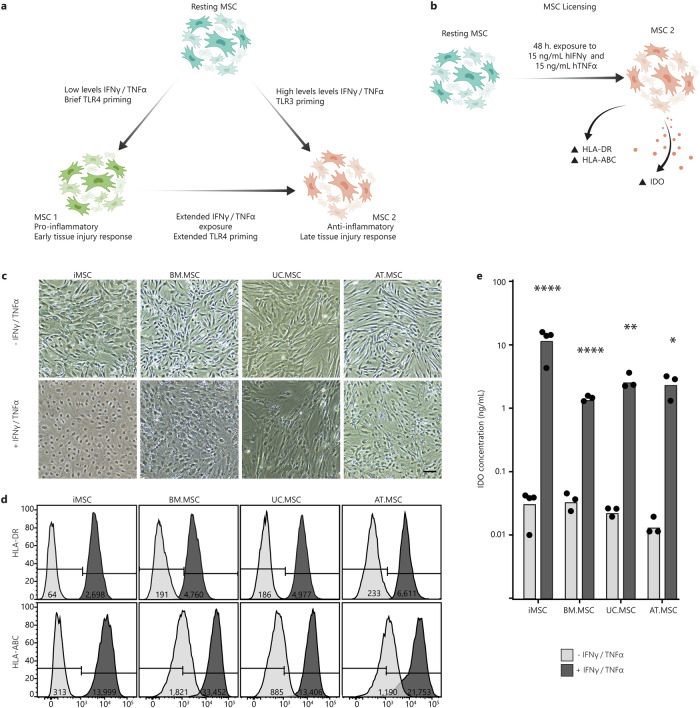
Inflammatory licensing of MSCs. **a** Schematic of the current model of MSC plasticity and MSC1/MSC2 licensing, created using Adobe Illustrator (2019). **b** An MSC2 phenotype was achieved by exposing resting MSCs to 15 ng/ml hIFNγ and 15 ng/ml hTNFα for 48 h and was characterised by increased expression of HLA-DR and HLA-ABC surface markers and secretion of IDO. **c** Brightfield images of MSCs from different sources with and without exposure to inflammatory cytokines. Scale bar is 25 µm. **d** Flow cytometry shift histograms of HLA-DR and HLA-ABC expression across MSCs from different sources with (+) (dark grey) and without (−) (light grey) inflammatory cytokines. Histograms are representative of one MSC line per source Median shift intensity is annotated within histograms. Data for replicate MSC lines are presented in Supplementary Fig. 3. **e** Bar chart of IDO concentration in CM from each MSC line with (+) (dark grey) and without (−) (light grey) inflammatory cytokines. Bars indicate mean IDO concentration across MSC lines with individual points for the mean of (*n* = 3 technical replicates) concentration for each MSC line. Statistical significance was calculated between each MSC line with and without exposure to cytokines by one-way ANOVA (*p < 0.0332, **p < 0.0021, ***p < 0.0002, ****p < 0.0001).

Previous studies have shown that MSC licensing can be initiated via stimulation with inflammatory cytokines, where short exposure times and lower concentrations result in an MSC1 phenotype, while higher concentrations or extended exposure result in an MSC2 phenotype^46–49^ (Fig. 1a). Here, an MSC2 phenotype was induced by exposure to 15 ng/ml IFN$\gamma$ and 15 ng/ml TNFα for 48 h, as per the ISCT recommendations on immune functional assays for MSCs^50^. Successful MSC2 licencing was validated by measuring the upregulation of HLA-ABC and HLA-DR surface markers and the secretion of IDO^10,15,50^ (Fig. 1b).

Upon exposure to the inflammatory cytokines, there were obvious changes to cell morphology, with visible membrane ruffling and reshaping of the cytoplasm (Fig. 1c). To validate that these changes were characteristic of inflammatory licensing, the expression of HLA-ABC and HLA-DR surface markers was measured using flow cytometry, both with and without cytokine exposure. In the absence of inflammatory cytokines (resting MSC state), all lines maintained an HLA-DR^neg^ phenotype, while the proportion of cells expressing HLA-ABC varied between MSC source. The percentage of HLA-ABC^pos^ cells in resting MSC lines was lowest in iMSCs (1.5–14%), followed by UC.MSCs (17–66%) and AT.MSCs (38–45%), and highest in BM.MSCs (50–80%) (Fig. 1d). In contrast, after exposure to inflammatory cytokines, >98% of MSCs expressed both HLA-DR and HLA-ABC, confirming that all the MSC populations had responded to the licencing procedure (licensed MSC state).

To subsequently determine whether the licenced MSCs had acquired an MSC1 or MSC2 phenotype, the level of IDO in MSC conditioned medium (CM) was assessed by ELISA. IDO plays a major role in the immunosuppressive functions that define the anti-inflammatory MSC2 phenotype and separate it from the MSC1 phenotype^10,15^. Exposure to inflammatory cytokines increased IDO levels in MSC CM by more than 10-fold for all MSC lines, indicating acquisition of an MSC2 phenotype. Importantly, while CM was produced from a standardised 1 × 10^4^ cells/mL of media, the concentrations of IDO varied considerably between sources. iMSCs secreted the most IDO (15.5-18 ng/mL), followed by UC.MSCs (2.5–4 ng/mL) and AT.MSCs (1.5–3.5 ng/ mL), with BM.MSCs producing the least (1.4–1.7 ng/mL) (Fig. 1e). Collectively, this indicated successful licencing of all MSCs lines after 48 h exposure to 15 ng/ml IFN$\gamma$ and 15 ng/mL TNFα, with a specific shift from a resting to MSC2 phenotype. These populations will subsequently be referred to as resting and licensed MSCs, respectively.

### Harvest of CM and quality assessment of MSC secretomes

To produce a comprehensive, unbiased proteomic profile of the MSC secretome, CM was harvested from both resting and licensed MSCs and LC-MS/MS was used to detect and quantify proteins. After stringent quality control to remove contaminants and media components, total protein lists were filtered against gene ontology cellular compartment (CC) term: *extracellular space* (GO:0005615) to identify secreted proteins. Total and extracellular protein lists are provided in Supplementary Data 1.

**Fig. 2:**
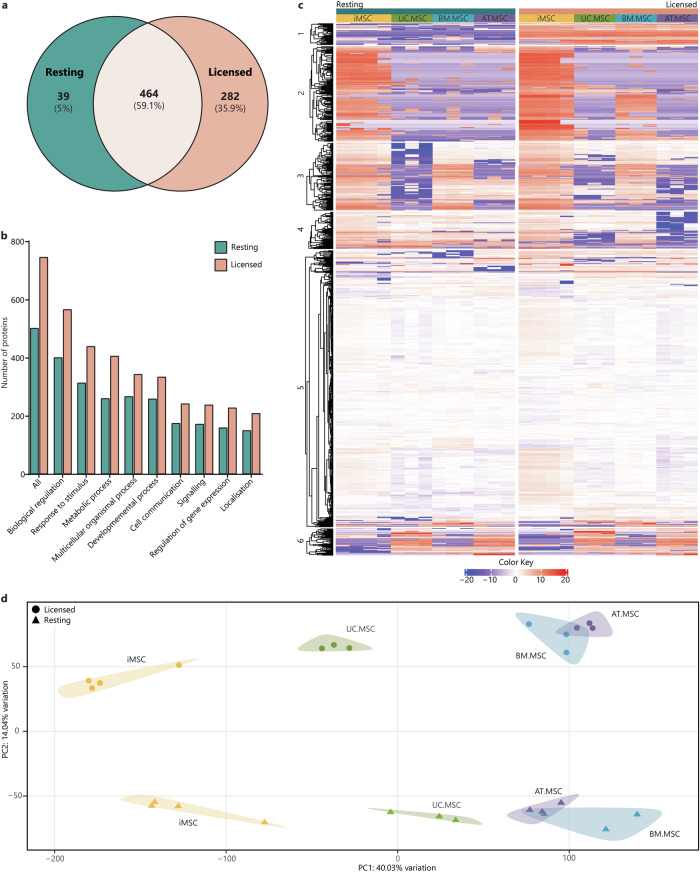
Characterisation of the MSC secretome by LC-MS/MS. **a** Venn diagram of the total number of secreted proteins in resting CM (teal) compared to licensed CM (coral). **b** Bar graph showing the numbers of proteins from resting (teal) and licensed (coral) CM annotated to the major GO biological process terms. **c** Heat map of proteomic profiles of MSC secretomes indicating protein intensity (red = more intense, blue = less intense). **d** PCA plot of relationships between MSC secretomes from different donors, sources and licensing phenotypes (iMSCs = yellow, UC.MSCs = green, AT.MSCs = purple, BM.MSCs = blue; resting MSCs = triangles, licensed MSCs = circles). Figures were generated using iDEP1.1^96^.

A total of 504 secreted proteins were identified in resting CM and 746 proteins were identified in licensed CM. Of these, 39 proteins (5%) were unique to resting conditions, 282 (35.8%) were unique to licensed conditions and the remaining 464 (59.2%) detected in both resting and licensed CM (Fig. 2a). Interestingly, a greater number of distinct proteins were identified in CM from iMSCs as compared to MSCs from other sources, regardless of whether the cells were in a resting or licensed state (Supplementary Fig. 4). Mapping of the secreted proteins to gene ontology biological process (BP) terms indicated that most of the proteins were linked to ‘*biological regulation*’, ‘*response to stimulus*’, and ‘*metabolic processes*’, with significant number of proteins involved in ‘*regulation of gene expression*’, ‘*general signalling’* and ‘*cell communication*’, regardless of whether they were present in resting or licenced CM (Fig. 2b). This emphasises the highly regulatory role that MSCs play under both resting and licensed conditions.

There were substantial differences between the MSC secretomes, demonstrated by varying patterns of protein intensity when comparing (i) resting and licensed MSCs, (ii) iMSCs and tissue-derived MSCs and (iii) MSCs from different tissue sources (Fig. 2c). Principal component analysis (PCA) was used to investigate the clustering and similarity of the MSC secretomes and showed clear effects of both inflammatory licencing and MSC source (Fig. 2d). Individual donors/batches from each combination of MSC source and treatment type clustered together, but there was distinct separation of the samples from different MSC sources and between those collected from cells in resting or licenced conditions. Principal component one (PC1) accounted for 40.0% of the variation and largely corresponded to differences due to MSC source. The greatest separation was observed between iMSC and tissue-derived MSC secretomes in general, with UC.MSC secretomes also separating from BM.MSC and AT.MSC secretomes along PC1, indicating greater similarity between the secretomes of iMSC and natal MSCs than between iMSC and adult tissue-derived MSCs. Principle component two (PC2) accounted for 14.0% of the variation of MSC secretomes and corresponded to separation of resting and licensed secretomes, which clearly formed into two distinct clusters.

### A conserved proteomic signature of licensed MSCs

**Fig. 3:**
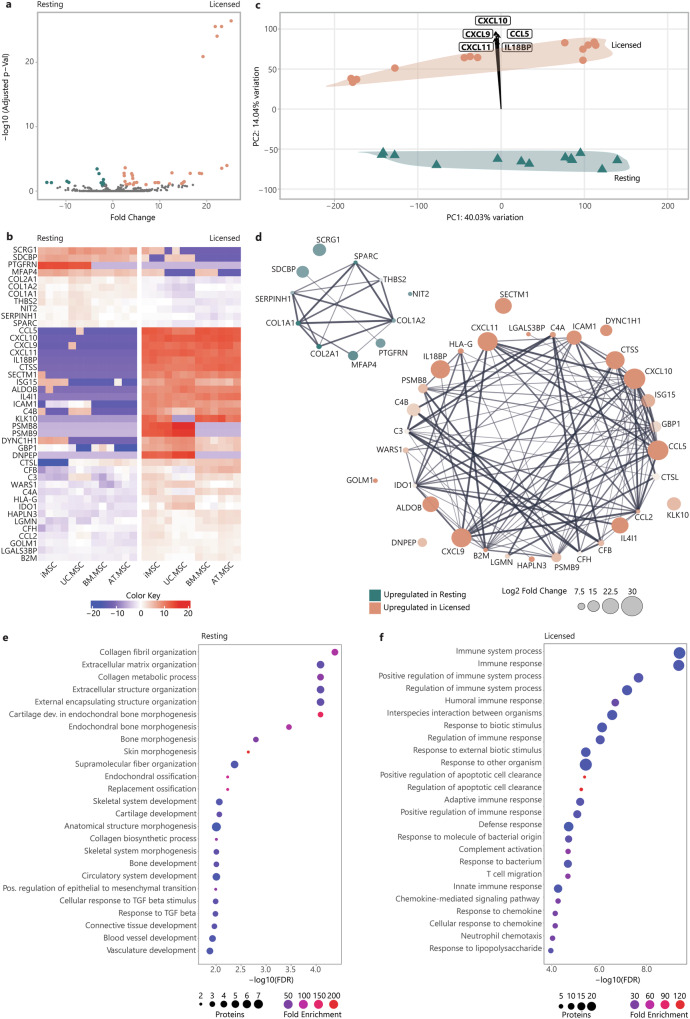
Comparison of resting and licensed MSC secretomes. **a** Volcano plot of fold change (x) versus statistical significance expressed as −log10(Adjusted *P* value) (y) of protein intensity between resting (teal) and licensed (coral) MSC secretomes. **b** Heatmap of protein intensity across MSC secretomes (x) (red = more intense, blue = less intense). **c** PCA loading plot showing specific proteins driving the separation of MSC sources along PC1 and resting (teal) vs licensed (coral) MSC secretomes along PC2. **d** Protein-protein interaction plot illustrating the strength of interactions between proteins DE between resting and licensed secretomes. Node colour indicates whether proteins are upregulated in resting (teal) or licensed (coral) secretomes, node size indicates fold change, node transparency indicates significance (adjusted *P* value), edge thickness indicates interaction confidence. Bubble plots of overrepresented biological process terms in resting (**e**) and licensed (**f**) secretomes. The -log10 FDR is plotted (x) with fold enrichment indicated by bubble colour (red = greater enrichment, blue = less enrichment), and the number of annotated proteins indicated by the bubble size. Figures were generated using iDEP1.1^96^ and Adobe Illustrator (2019).

The changes that MSCs undergo with inflammatory licensing, and the effect that this has on their secretome, represent a crucial gap in our understanding of MSC behaviour. To identify a conserved signature of inflammatory licensing in the MSC secretome, differential expression (DE) of proteins were calculated between paired resting and licenced MSC lines. This detected 43 DE proteins that were shared between resting and licensed secretomes, regardless of MSC source or donor. Of these, 32 proteins were upregulated in licensed secretomes and 11 were upregulated in resting secretomes (Fig. 3a). DE of all secreted proteins is provided in Supplementary Data 1.

DE proteins included known drivers of key MSC functions, such as IDO, which inhibits the conversion of tryptophan into kynurenine, thereby inducing T cell anergy^10^; prostaglandin E-synthase (PTGES3) which is involved in the synthesis of prostaglandin E, which plays a role in inflammation and immunomodulation^42^; TNFα stimulated gene-6 (TSG-6), which suppresses inflammation by inhibiting the NF-κB signalling pathway^51,52^; monocyte chemoattractant protein 1 (CCL2), which recruits monocytes to sites of inflammation, aiding in tissue repair^53^; interleukin 6 (IL-6), which promotes B cell maturation and acts as a pro-inflammatory mediator; and, HLA-A, B, and C which are critical for presenting antigenic peptides to T cells, facilitating immune recognition. Less predictably, cathepsin S (CTSS) contributes to the degradation of proteins in the lysosome as well as effecting cell migration and homing. This underscores the considerable influence that licensing of MSCs has on their function. Additionally, proteins not previously linked to inflammatory licencing were also identified as DE.These included: laminin subunit alpha 2 (LAMA2), a component of the ECM that supports cell adhesion and migration; aldolase B (ALDOB); and, midkine (MDK), a heparin-binding growth factor that promotes cell proliferation and migration (Fig. 3b). These DE proteins represent a conserved signature of the secretome of resting and licensed MSCs that will provide important fundamental insights into the nature of the different MSC states and their functional differences.

To verify the main drivers of the separation of resting and licensed secretomes (PC2), PCA loadings were calculated (Fig. 3c). These showed that the principal component separating resting and licensed MSC secretomes was primarily driven by chemokine (C-X-C motif) ligand 9 (CXCL9), CXCL10, CXCL11, chemokine (C-C motif) ligand 5 (CCL5) and interleukin-18 binding protein (IL18BP), all of which were upregulated by over 20-fold in the licenced secretomes. In contrast, proteins that were more abundant in the resting MSC secretome included collagens (COL)1A1, COL1A2, COL2A1, microfibril-associated glycoprotein 4 (MFAP4), prostaglandin F2 receptor negative regulator (PTGFRN), thrombospondin-2 (THBS2) and syndecan-binding protein (SDCBP). Significantly DE proteins between paired resting-licensed secretomes are included in (Supplementary Table 2).

To better understand the different functional roles that the secretomes of resting and licensed MSCs may play, the STRING database^54^ was used to generate interaction networks between proteins upregulated in resting or in licensed secretomes (Fig. 3d). This identified highly interconnected networks, with relatively few disconnected proteins, for each the resting and licensed secretomes, suggesting strong functional overlap within resting and licensed secretory profiles. To explore this, and the biological impact of secretory profile changes, DE proteins were interrogated for statistically over-represented BP using GO-term enrichment analysis. When analysing the most significant processes, those with the lowest false discovery rate (FDR), it emerged that proteins enriched in resting secretomes were overrepresented in processes linked to ECM deposition and remodelling, including ‘*collagen fibril organisation’* (90.0-fold), ‘*extracellular matrix organization’* (24.7-fold), ‘*bone morphogenesis’* (52.1-fold), ‘*skin morphogenesis’* (219.3-fold), ‘*replacement ossification’* (121.0-fold), ‘*skeletal system development’* (12.5-fold), ‘*cartilage development’* (24.8-fold), ‘*circulatory system development’* (7.6-fold) and *‘response to TGFβ’* (19.4-fold) (Fig. 3e). In contrast, proteins enriched in the licensed MSC secretomes were predominantly linked to processes involved in immune regulation, including ‘*immune system process’* (4.5-fold), ‘*immune response’* (5.8-fold), ‘*positive regulation of immune system process’* (8.1-fold), ‘*humoral immune response’* (18.0-fold), ‘*adaptive immune response’* (9.3-fold), and ‘*complement activation’* (35.9-fold) (Fig. 3f) (Table 1). This emphasises the pro-regenerative properties of the resting MSC secretome in contrast to the immunomodulatory properties of the MSC2 secretome. Moreover, it suggests that pro-regenerative properties of the resting MSC secretome are heavily downregulated with inflammatory licensing.

**Table 1:** Top biological process terms overrepresented amongst DE proteins upregulated in either resting or licensed MSC secretomes

Group	Biological process	Fold enrichment	FDR
*Overrepresented in licensed*	Immune system process	4.55	3.69E-10
	Immune response	5.78	3.69E-10
	Positive regulation of immune system process	8.08	2.90E-08
	Regulation of immune system process	5.91	8.81E-08
	Humoral immune response	17.97	2.79E-07
	Biological process involved in interspecies interaction between organisms	5.35	2.79E-07
	Response to biotic stimulus	5.45	7.45E-07
	Regulation of immune response	7.44	8.95E-07
	Response to external biotic stimulus	5.23	3.64E-06
	Response to other organism	5.23	3.64E-06
	Positive regulation of apoptotic cell clearance	120.61	4.10E-06
	Regulation of apoptotic cell clearance	109.65	5.66E-06
	Adaptive immune response	9.35	6.31E-06
	Positive regulation of immune response	9.00	8.36E-06
	Defence response	4.45	1.93E-05
	Response to molecule of bacterial origin	12.06	1.94E-05
	Complement activation	35.90	2.04E-05
	Response to bacterium	7.81	2.08E-05
	T cell migration	35.06	2.08E-05
	Innate immune response	6.02	5.40E-05
	Chemokine-mediated signalling pathway	28.45	5.40E-05
	Response to chemokine	26.22	7.14E-05
	Cellular response to chemokine	26.22	7.14E-05
	Neutrophil chemotaxis	24.51	9.25E-05
	Response to lipopolysaccharide	11.23	1.13E-04
*Overrepresented in resting*	Collagen fibril organisation	89.97	4.84E-05
	Extracellular matrix organisation	24.71	9.24E-05
	Collagen metabolic process	64.38	9.24E-05
	Extracellular structure organisation	24.64	9.24E-05
	External encapsulating structure organisation	24.43	9.24E-05
	Cartilage development involved in endochondral bone morphogenesis	159.49	9.24E-05
	Endochondral bone morphogenesis	89.20	3.82E-04
	Bone morphogenesis	52.11	1.69E-03
	Skin morphogenesis	219.30	2.38E-03
	Supramolecular fibre organisation	9.93	4.41E-03
	Endochondral ossification	120.99	6.02E-03
	Replacement ossification	120.99	6.02E-03
	Skeletal system development	12.49	8.76E-03
	Cartilage development	24.83	8.76E-03
	Anatomical structure morphogenesis	4.28	1.00E-02
	Collagen biosynthetic process	76.28	1.00E-02
	Skeletal system morphogenesis	22.49	1.00E-02
	Bone development	21.39	1.00E-02
	Circulatory system development	7.63	1.00E-02
	Positive regulation of epithelial to mesenchymal transition	67.48	1.03E-02
	Cellular response to transforming growth factor beta stimulus	19.86	1.03E-02
	Response to transforming growth factor beta	19.42	1.05E-02
	Connective tissue development	18.60	1.10E-02
	Blood vessel development	9.56	1.20E-02
	Vasculature development	9.17	1.35E-02
	Cellular response to amino acid stimulus	46.17	1.66E-02

To better understand the regulatory mechanisms that drive the differences between resting and licensed MSC phenotypes, DE protein lists were further queried for overrepresentation in specific transcription factor (TF) regulatory networks using the hTFtarget database^55^. Regulatory networks governing the expression of proteins upregulated in resting MSC secretomes were enriched for TFs involved in developmental processes, including SP3 (32.8-fold), KLF13 (442.7-fold) and SOX4 (885.4-fold). In contrast, regulatory networks governing expression of proteins upregulated in licensed MSC secretomes were enriched for known drivers of immune responses, including STAT1 (7.8-fold), STAT2 (7.0-fold), REL (10.8-fold), RELA (7.8-fold), RELB (23.9-fold), NFkB1 (5.6-fold) and NFkB2 (9.9-fold) targets, as well as IRF1 (8.7-fold), IRF3 (82.5-fold) and CEBPD (45.3-fold) (Table 2). This suggests that the resting MSC phenotype is maintained by activity of a limited number of TFs which play important roles in regulating developmental processes and tissue homoeostasis while the change in secretory profiles with inflammatory licensing is driven by activation of IRF and Rel family TFs, which are known regulators of inflammatory processes^52,56–59^.

**Table 2:** Overrepresented transcription factor target regulatory networks of proteins enriched in licensed MSC secretomes

	TF regulatory networks	Fold enrichment	Adjusted *P*- values
*Overrepresented in resting*	SP3	32.8	2.63E-2
	KLF13	442.7	2.63E-2
	SOX4	885.4	2.63E-2
*Overrepresented in licensed*	STAT1	7.8	1.60E-5
	IRF3	82.5	2.89E-4
	RELA	7.8	4.12E-4
	RELB	23.9	4.12E-4
	NFKB2	9.9	4.12E-4
	REL	10.8	1.25E-3
	NFKB1	5.6	1.92E-3
	STAT2	7.0	1.92E-3
	CEBPD	45.3	9.22E-3
	IRF1	8.7	9.68E-3

Overall, these data provide a conserved signature of the MSC secretome in resting and MSC2 states. Key changes between these proteomic profiles suggest that the role of resting MSCs focuses heavily on tissue maintenance and repair, through the deposition of ECM proteins and the promotion of developmental, vascular and morphogenic processes. In contrast, under inflammatory licensed conditions, these functions are strongly downregulated, in favour of the secretion of a complex milieu of immunomodulatory proteins. Importantly, these secretory profiles are generally conserved between iPSC and tissue-derived MSCs indicating that iMSCs undergo a comparable phenotypic plasticity to tissue-derived MSCs, which is key to establishing their functional equivalence as immunomodulatory therapies.

### Differences in the secretome of MSCs by cell source

While clear differences between the secretomes of resting and licensed MSCs showed the impact of inflammatory licencing (PC2,14.0% of variation), significantly more of the variation between the secretomes was due to the MSC source (PC1, 40.0% of variation). While most of these differences were specific to either resting or licensed MSCs, it is noteworthy that a small subset of proteins drove the separation of all MSC sources along PC1 regardless of whether the cells were in a resting or licensed state. These were profilin-2 (PFN2), eukaryotic translation initiation factor 2 subunit 1 (EIF2S1) and high mobility group box 1 (HMGB1), which were highest in iMSC secretomes and lowest in BM.MSC and AT.MSC secretomes (Supplementary Fig. 5a–c). Conversely, cystatin-2 (CST2) and proenkephalin (PENK) were enriched in adult tissue-derived MSCs and lowest in iMSCs (Supplementary Fig. 5d, e). Expression of these proteins likely represents a singular conserved variable driving source-based heterogeneity of MSCs regardless of functional state.

**Fig. 4:**
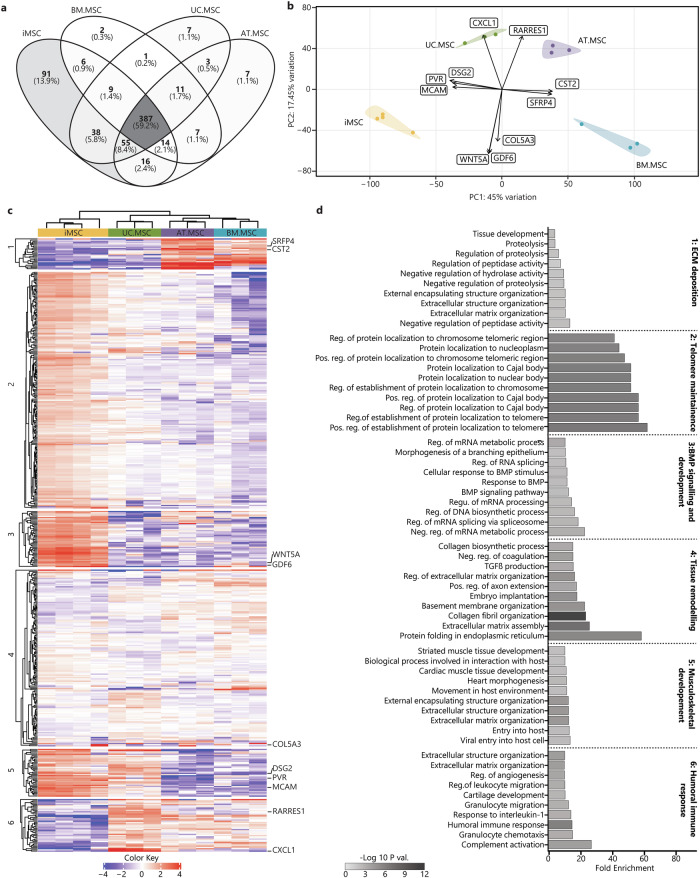
Heterogeneity of the resting MSC secretome between MSC sources. **a** Venn diagrams represent the total amount of identified proteins in CM from different sources (*n* = 4 iMSC, *n* = 3 BM.MSC, *n* = 3 UC.MSC, *n* = 3 UC.MSC), where more exclusive proteins were found in iMSC secretomes. **b** PCA loadings were plotted identifying specific proteins driving the separation of resting MSC secretomes by sources (iMSC = yellow, BM.MSC = blue, AT.MSC = purple, UC.MSC = green). **c** Heat map showing hierarchical Euclidean (average) clustering of resting MSC secretomes (x) and K-means clustering (*n* = 6) of secreted proteins (y) based on intensity (red = more intense, blue = less intense). **d** Top 10 overrepresented GO BP terms for each K-means cluster of resting MSC secretomes were plotted as horizontal bar graphs. Bar length indicates fold enrichment, colour indicates significance (−log10 FDR) as per the legend. Figures were generated using iDEP1.1^96^.

Within the resting secretomes, 387 proteins (59.2%) were conserved between all MSC sources. The iMSC secretomes were most different of all the samples and contained 91 unique proteins, while the UC.MSC and AT.MSC secretomes each contained seven unique proteins, and the BM.MSC secretomes just two unique proteins (Fig. 4a). Using the coefficient of variation (CoV) to indicate the homogeneity of the secretomes within each source, it was evident that there was less variability between different iMSC batches (CoV 18%) than between different donors of BM.MSCs (CoV 41%), UC.MSCs (CoV 26%) or AT.MSCs (CoV 24%) (Supplementary Fig. 6a). PCA was used to visualise the overall similarity between the secretomes and showed that separate donors/batches of MSCs from the same source clustered together, while different sources were separated. The MSC secretomes separated by source along PC1 (45% of overall variation) with BM.MSCs plotting on the most positive end of PC1, followed by AT.MSCs, UC.MSC and finally iMSCs, which plotted at the most negative end (Fig. 4b). This axis of separation, with iMSC and UC.MSC secretomes on one end and adult-tissue derived secretomes on the other end, was driven by CD155 (poliovirus receptor/PVR), melanoma cell adhesion molecule (MCAM) and desmoglein 2 (DSG2), which were higher in iMSC and UC.MSC secretomes; and secreted frizzled-related proteins 4 (SFRP4) and cystatin 2 (CST2) which were strongest in BM.MSC and AT.MSC secretomes (Supplementary Fig. 7a–e). The other principal component of variability (PC2, 17.45% of variation) showed separation of UC.MSC and AT.MSC secretomes from the iMSC and BM.MSCs and was driven by increased CXCL1 and retinoic acid receptor responder 1 (RARRES1), and reduced COL5A3, Wnt family member 5A (WNT5A) and growth differentiation factor (GDF6) in UC.MSC and AT.MSC secretomes (Supplementary Fig. 8a–e).

To further identify patterns in the expression level of proteins secreted by MSCs from different sources, two-way heat maps were generated, and K-means clustering was used to group proteins with similar expression patterns, forming six specific clusters (Fig. 4c). Consistent with the PCA, the heatmap clustering showed the iMSC secretome to be distinct from that of tissue-derived MSCs. Of these, UC.MSCs bore the most similarity to the iMSCs, whilst the AT.MSC and BM.MSC were very different to the iMSCs but had profiles that were closely aligned to each other. To determine whether the proteins within the six clusters had shared biological functions, they were queried for enrichment of BP terms. Clusters one and five contained proteins that segregated iMSCs and UC.MSCs from AT.MSCs and BM.MSCs. The proteins in cluster one were higher in AT.MSC and BM.MSCs, and were overrepresented in fibrotic processes including: ‘*extracellular matrix organisation’*, ‘*regulation of proteolysis’*, and ‘*tissue development’*, whilst those in cluster five were lower in AT.MSC and BM.MSCs and were overrepresented in developmental processes such as ‘*heart morphogenesis’* and ‘*striated muscle tissue development’*. The proteins in clusters two and three were strongest in iMSC secretomes and were overrepresented in processes indicating stem cell-like properties and telomerase activity, including: ‘*regulation of protein localization to Cajal body’*, ‘*positive regulation of establishment of protein localization to telomere’*, and ‘*positive regulation of protein localization to chromosome telomeric region’*. In contrast proteins in cluster six, which were enriched in tissue-derived, but not iMSC, secretomes, were overrepresented in both pro-inflammatory and skeletal tissue developmental processes, including: ‘*complement activation’*, ‘*humoral immune response’*, ‘*cartilage development’* (Fig. 4d) (Supplementary Table 3). The variation in functional enrichment between the secretomes of MSCs from different sources highlights the more stem-cell like and developmental identities of iPSC and UC.MSCs which contrasts with the more structural and homoeostatic identities of adult tissue-derived MSCs, highlighting the need to carefully consider MSC source to effectively target desired outcomes.

**Fig. 5:**
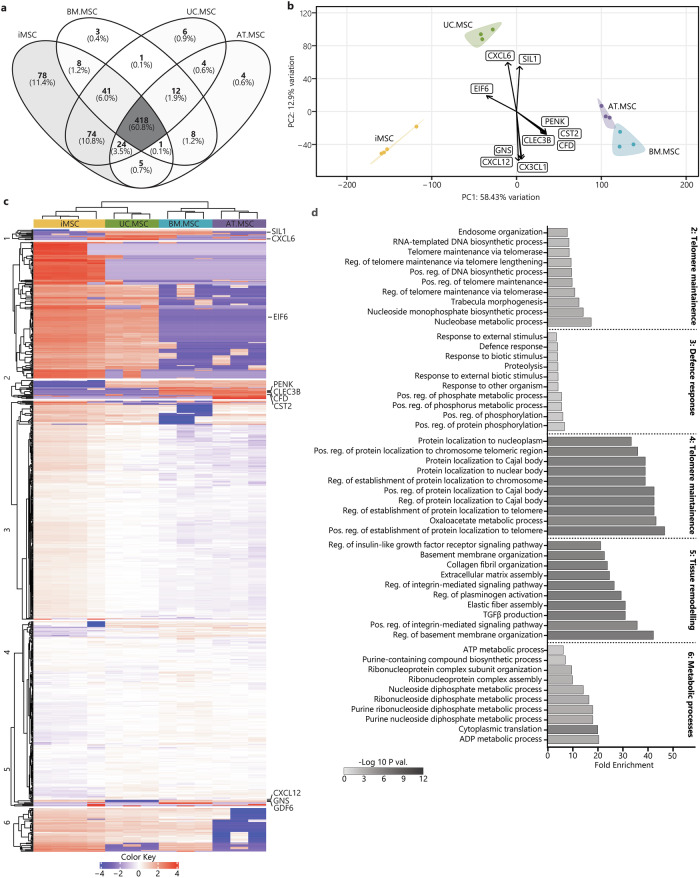
Heterogeneity of the licensed MSC secretome between MSC sources. **a** Venn diagrams represent the total amount of identified proteins in CM from different sources (*n* = 4 iMSC, *n* = 3 BM.MSC, *n* = 3 UC.MSC, *n* = 3 UC.MSC), where more exclusive proteins were found in iMSC secretomes. **b** PCA loadings were plotted identifying specific proteins driving the separation of licensed MSC secretomes by sources (iMSC = yellow, BM.MSC = blue, AT.MSC = purple, UC.MSC = green). **c** Heat map showing hierarchical Euclidean (average) clustering of licensed MSC secretomes (x) and K-means clustering (*n* = 6) of secreted proteins (y) based on intensity (red = more intense, blue = less intense). **d** Top 10 overrepresented GO BP terms for each K-means cluster of licensed MSC secretomes were plotted as horizontal bar graphs. Bar length indicates fold enrichment, colour indicates significance (−log10 FDR) as per the legend. Figures were generated using iDEP1.1^96^.

The data was then interrogated to determine how MSC source affects the composition of the secretome after inflammatory licencing. Across the licensed secretomes, 418 proteins (60.8%) were conserved between all sources, which was very similar to the 59.2% conservation measured in resting secretomes. Also bearing similarity to the resting MSCs, the licenced iMSCs secretomes were most distinct, containing 78 unique proteins, followed by UC.MSC secretomes with six unique proteins, AT.MSC secretomes with four unique proteins, and BM.MSC secretomes with just three unique proteins (Fig. 5a). The coefficients of variation (CoV) between licensed MSCs also showed less variability between the batches of iMSC secretomes (CoV 17%) than observed for secretomes from different donors of BM.MSCs (CoV 40%), UC.MSCs (CoV 30%) or AT.MSCs (CoV 44%) (Supplementary Fig. 6b).

PCA of the licensed secretomes showed a similar pattern to the resting samples, with greater similarity between the iMSC and UC.MSC secretomes, and separation of these from BM.MSC and AT.MSC secretomes along PC1 (55.36% of overall variation) (Fig. 5b). This separation of iMSC and UC.MSC secretomes from the adult-tissue derived secretomes was driven by reduced expression of PENK, CST2, complement factor D (CFD) and C-type lectin domain family 3 member B (CLEC3B), and increased levels of eukaryotic translation initiation factor 6 (EIF6), which was highest in the iMSC followed by UC.MSC secretomes (Supplementary Fig. 9a–e). The second principal component of variability (PC2, 13.18% of variation) separated the UC.MSC secretomes from all other cell sources and was driven by increased CXCL6 and decreased CXCL12, CX3CL1 and glucosamine (N-acetyl)-6-sulfatase (GNS). Interestingly, UC.MSC and BM.MSC secretomes, as well as commercial but not clinical-grade iMSC secretomes shared concentrations of nucleotide exchange factor SIL1 (SIL1), a processing protein that has been previously identified at a transcriptomic level in certain sub-populations of MSCs^60^ (Supplementary Fig. 10a–e).

Hierarchical clustering of the proteomic profiles supported the PCA findings, showing the greatest similarity between BM.MSC and AT.MSC secretomes and separation of these from the iMSC and UC.MSC secretomes. As was done for the resting secretome, proteins were grouped into six K-means clusters to identify expression patterns and uncover distinct functional modules within the licensed secretome of MSCs from different sources (Fig. 5c). The biological functions of these K-means clusters were explored by GO enrichment analysis of BP terms. Cluster one consisted of 11 proteins which were highest in UC.MSC secretomes, likely responsible for the separation of UC.MSC secretomes from those of other MSC lines along PC2. While this represents a unique signature of the licensed UC.MSC secretome, the functions of these proteins did not converge on any particular process and no BP terms were significantly overrepresented by these proteins. Clusters two and four included proteins most strongly expressed in iMSC and UC.MSC secretomes, with the proteins in cluster two being more strongly upregulated than those in cluster four. The proteins in these clusters were likely responsible for the separation of these from the AT.MSC and BM.MSC secretomes and were overrepresented in processes indicating stem/progenitor-cell like properties and telomerase activity, with those in cluster two including ‘*regulation of telomere maintenance via telomerase’*, and ‘*telomere maintenance via telomerase’*, while those in cluster four were overrepresented in *oxaloacetate metabolic process* and multiple processes pertaining to the ‘*regulation of protein localization to telomere and Cajal body’*. Notably, the maintenance of telomere length is crucial for sustained cell division and the delay of senescence. It is a characteristic feature of highly proliferative stem cell populations and suggestive that the strong regenerative potential of both iMSC/UC.MSCs, but not BM.MSC/AT.MSCs, is maintained under both resting and licensed conditions^61^. Proteins in cluster five were weakly enriched in tissue-derived secretomes compared to iMSCs and were overrepresented in process linked to cell adhesion and ECM, including ‘*positive regulation of integrin mediated signalling’, ‘extracellular matrix assembly’* and ‘*transforming growth factor beta 1 production’*. Finally, proteins in cluster three, which were upregulated in BM.MSC and AT.MSC secretomes, were overrepresented in ‘*defence response’*, ‘*response to other organism’* and ‘*positive regulation of protein phosphorylation’* (Fig. 5d) (Supplementary Table 4). This suggests that, despite the largely conserved secretory profile of licensed MSC secretomes, there are still differences in expression of specific factors between MSCs from different sources. Overall, however functional enrichment indicates that across licensed MSCs secretomes, most of the source-based variation continues to be driven by the activity of telomerase in iMSC, and to a lesser extent UC.MSCs, and the secretion of matrix proteins by AT.MSC and BM.MSCs.

### Comparison of functional effects of resting and licensed MSC CM

To assess whether the proteomic profiles of resting and licensed MSC secretomes were predictive of their functional effects we characterised the effect of MSC CM using in vitro models of wound healing, angiogenesis, and immunomodulation.

**Fig. 6:**
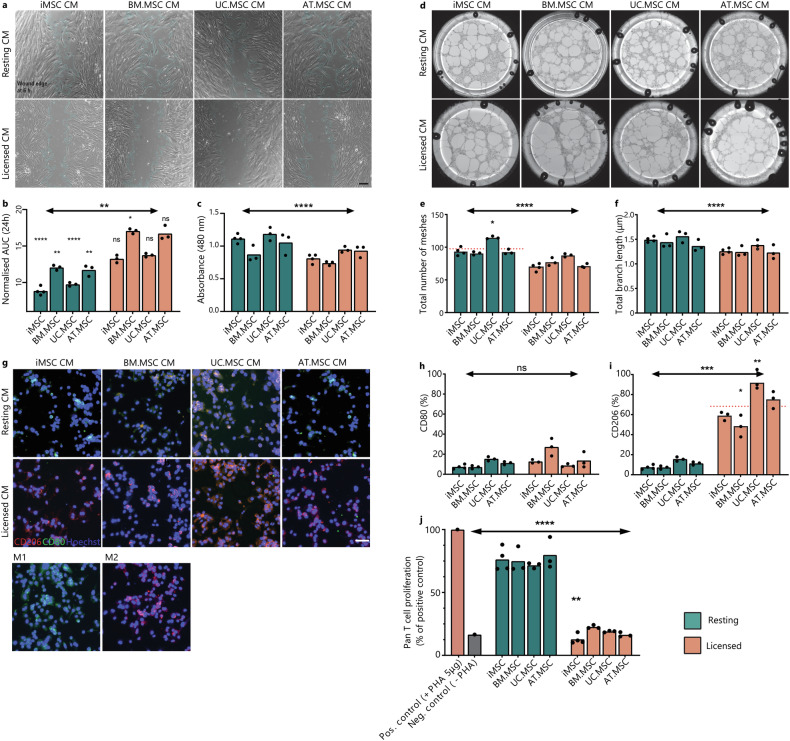
Functional response to MSC CM. **a** Representative images of wounded fibroblast cultures at 6 h. in resting or licensed CM from different MSC sources. Scale bar is 200 μm. Wound edge is outlined in teal using ImageJ Wound Healing Analysis plugin. **b** Bar graph showing the area under the curve (AUC) of wound size in a fibroblast monolayer treated with resting or licensed CM from different MSC sources in comparison to fresh media control (grey). **c** Bar graph showing the absorbance of MTS (490 nm) as a proxy for fibroblast number after 5 days culture in resting or licensed CM from different MSC sources. **d** Representative images of endothelial tube formation assays at 18 h. in resting or licensed CM from different MSC sources. Diameter of well is 39 mm. Bar graph showing the total number of meshes (**e**) and total branching length (μm) (**f**) of angiogenic networks. ImageJ AngiogenicAnalyser plugin was used to quantify network components. **g** Representative images of macrophage cultures treated with resting or licensed CM from different MSC sources for 48 h. showing CD206 (red), CD80 (green) and nuclei (blue). Control Macrophage cultures were treated with IFNy/LPS (M1) or IL-4/IL-13 (M2) for 48 h. Scale bar is 50 μm. Bar graphs quantifying the CD80+ (**h**) and CD206+ (**i**) populations of macrophages identified by flow cytometry. **i** Bar graph showing the proliferation of activated pan T-cells treated with resting or licensed CM from different MSC sources (1:2 ratio) in comparison to activated (+PHA) or inactivated (−PHA) (±5μg/mL PHA) controls. For bar graphs (**b**, **c**, **e**, **f**, **h**–**j**), bar height shows the mean of (*n* = 3) biological replicates with individual points showing the mean of (*n* = 3) technical replicates for each MSC line under resting (teal) or licensed (coral) conditions. Statistical significance was calculated by one-way ANOVA (*p < 0.0332, **p < 0.0021, ***p < 0.0002, ****p < 0.0001) against fresh media control (*n* = 3 technical replicates).

The proteomic data indicated increased levels of mitogens, matrix proteins and adhesion molecules in resting vs licensed CM which would suggest that CM from resting MSCs is better suited to promote wound healing processes. To test this, the proliferation and migration of dermal fibroblasts was tested via in vitro scratch assay (Fig. 6a). As predicted by the proteomic profiles, resting MSC CM from all sources significantly reduced cumulative wound size compared to fresh media. This was not observed in cultures treated with licensed CM where licensed BM.MSC CM actually delayed wound closure when compared to unconditioned controls. Under resting conditions, calculation of cumulative wound closure showed that both iMSC and UC.MSC CM resulted in faster wound closure than either BM.MSC or AT.MSC CM (*p* = <0.001) (Fig. 6b). To separate the effects of proliferation and migration on wound closure, proliferation of fibroblasts was assessed separately by MTS assays. In comparison to licensed CM, resting CM significantly increased fibroblast proliferation (*p* = 0.003). This response was strongest with treatment from resting iMSC and UC.MSC (Fig. 6c). Similarly, when migration distance across the wound site was calculated, cells treated with resting MSC CM migrated 1.5–4.0 fold faster than those treated with corresponding licensed MSC CM (Supplementary Fig. 11).

As resting MSC secretomes were also enriched for proteins linked to vascular development and angiogenic processes, the vascular response of endothelial cells was tested (Fig. 6d). Resting MSC CM resulted in faster and more stable tube formation, significantly higher mesh area (*p* = <0.0001) (Fig. 6e), and branch length (Fig. 6f). Of particular note, resting UC.MSC secretomes supported the greatest mesh formation, more than 25% higher than resting MSC CM from other sources. Upon closer investigation of expression patterns of proteins annotated to vascular development, this corresponded to uniquely high secretion of Vascular Endothelial Growth Factor C (VEGFC) and the corresponding receptor, Neuropilin 2 (NRP2) (Supplementary Fig. 12). VEGFC/NRP2 are well established drivers of in vitro and in vivo angiogenesis^62^, supporting the capacity for this data set to be used to predict optimal MSC sources for specific target applications.

While resting MSCs were enriched for pro-regenerative factors, licensed MSC secretomes were enriched for both humoral and innate immunomodulatory proteins. To assess the differential effects of resting and licensed MSC CM, activated monocyte populations were cultured in either resting or licensed MSC CM and the polarisation of macrophages to a pro-inflammatory M1 or an anti-inflammatory M2 phenotype was characterised. Treatment with resting MSC CM did not result in significant polarisation of monocytes to either a M1 or an M2 macrophage phenotype, with limited expression of either CD80 or CD206 detected by immunofluorescence or flow cytometry. In contrast, treatment with licensed MSC CM resulted in a strong shift towards M2 macrophages (Fig. 6g, h). Across licensed secretomes, treatment with UC.MSC CM resulted in the strongest polarisation, with greater than 90% of treated macrophages expressing CD206+. In contrast, BM.MSC CM produced the weakest response, with an average of 47% CD206+ cells (Fig. 6i). By comparing these response patterns with proteomics data, we identified that both transforming growth factor-1 and 2 (TGFβ1/2) and C-C Motif Chemokine Ligand 2 (CCL2) intensity in licensed MSC CM corresponded with the ability to induce M2 polarisation of macrophages, with higher secretion in licensed secretomes and most strongly by iMSC and UC.MSC populations.

Finally, the ability of MSCs to inhibit proliferation of activated T cells was also tested. This is a key aspect of their immunomodulatory identity and central to their therapeutic potential in autoimmune diseases^63^. Activated pan T cells were treated with resting or licensed CM from the different MSC sources. Regardless of source, the resting CM failed to significantly inhibit T cell proliferation, indicating that without adequate inflammatory licensing MSCs do not produce the necessary factors to perform this function (Fig. 6j). In contrast, the licensed CM significantly inhibited T cell proliferation regardless of MSC source (*p* = <0.0001). A comparison between MSC sources showed that iMSC CM more effectively reduced T cell proliferation than any tissue-derived MSC CM, evidenced by an 11.7% reduction from BM.MSC CM, 8.1% from UC.MSC CM, and 5.5% from AT.MSC CM. This ability to inhibit T cell proliferation correlated strongly with the acquisition of an MSC2 phenotype. It also mirrored the amount of IDO produced, which was only detected at significant levels under licensed conditions and which was higher in iMSC secretomes compared to BM.MSC secretomes.

Collectively, these results support the hypothesis that MSCs exhibit a strong phenotypic switch upon inflammatory licensing, resulting in robust changes to their secretory profiles which subsequently predict their functional effectiveness in key responder cell assays. Overall, global proteomic profiles or resting and licensed MSCs correlated strongly with the ability of resting MSC CM to most effectively promote wound closure and angiogenic tube formation while licensed MSC secretomes induced M2 polarisation of macrophages and inhibited proliferation of activated T cells.

## Discussion

This study used high resolution two-dimensional LC-MS/MS to profile the complete secretory profiles of both resting and licensed MSCs across multiple tissue sources, donors and iPSC-platforms. Previously, differences between the secretomes of MSCs from different sources have only ever been thoroughly described under resting conditions^45,64,65^ while descriptions of factors secreted by licensed MSCs have been restricted to small numbers of specific proteins^52,63,66^, and often only from a single tissue type or donor^65,67^. By analysing paired resting and licensed secretomes from bone marrow, adipose tissue, umbilical cord and iPSC-derived MSCs, this study provides a uniquely comprehensive view of how MSC source and phenotype interact to shape the secretory profile. This pairwise-comparative approach has also allowed us to recognise a core signature of MSC licensing that is conserved across donors and tissue sources and identify source-dependent variations in both resting and licensed phenotypes. Importantly, these proteomic profiles also demonstrate for the first time that immunomodulatory plasticity and the MSC2 phenotype are recapitulated by iPSC-derived MSCs. This fills a critical gap in our understanding of MSC biology and yields new insights into the defining criteria of these cells.

The selection of bone marrow, adipose, and umbilical cord-derived MSCs incorporates the most commonly used tissue-sources and provides insights into differences between MSCs derived from adult and natal tissues (i.e. varying in vivo age). Additionally, by including MSCs from multiple donors/batches, our dataset enabled conclusions to be made regarding the consistency of the secretome in relation to donor or batch variability. Furthermore, the inclusion of independent batches of clinical-grade iMSCs alongside a commercially available iMSC line also permitted investigation into the potential differences between iMSC batches, something that was previously unknown. Importantly though, while biological triplicates are considered adequate for the characterisation of different MSC populations^68–70^ the significant donor-donor variation observed here, particularly between adult tissue-derived MSCs, highlights the need for additional studies exploring the complete scope of donor-donor variation within both tissue and iPSC-derived MSC populations including additional clinical iMSC products as they become available.

All MSCs were cultured in parallel to passage six to allow sufficient expansion of tissue-derived MSCs and align them with clinical iMSC batches, which are administered after passage five. At this point, all MSC lines were confirmed to have maintained a fibroblastic morphology and proliferative capacity, indicating that they had not reached senescence. They also adhered to the ISCT minimal criteria, expressing appropriate surface antigen profiles and demonstrating in vitro tri-lineage differentiation. Whilst all the MSC lines included in this study met the ISCT minimal criteria^3^, there were still stark differences in their secretory profiles and immunomodulatory potential which reinforces the need to understand the differences between MSC sources and the potential therapeutic impact this may have.

While MSC plasticity is central to both their in vivo identity and their therapeutic potential, only a limited number of studies have previously addressed this, leaving much to be understood about the process of MSC licensing and the differences between the different MSC subtypes. Specifically, prior to this study there was no information available as to if, or how, iMSCs responded to inflammatory microenvironments and it was unknown whether the secretomes of iMSCs would possess comparable immunomodulatory properties to tissue-derived MSCs. In this study, all MSC lines, including both clinical and commercially available iMSCs, licensed to an MSC2 immunosuppressive phenotype after 48 h exposure to 15 ng/mL IFNγ and 15 ng/mL TNFα. Following the currently accepted markers for MSC licencing, this was verified by upregulation of HLA-DR and HLA-ABC surface markers and the increased secretion of IDO^11,15,16^. These indicators are well characterised across tissue-derived MSCs^71–75^ but have not previously been demonstrated in iMSCs. The demonstration of phenotypic plasticity by iPSC-derived MSC populations is hugely important for their successful application as a cell therapy as this process underpins the immunomodulatory properties of MSCs and the mechanisms by which they are thought to act in many of their target applications^76^. This comparable response to inflammatory factors also validates the equivalency of iMSCs to tissue-derived MSCs in one of the most fundamental and complex MSC behaviours, and further supports the potential for iMSCs to be applied in many of the contexts where tissue-derived MSCs have shown promise. Considering the MSC panel as a whole, the observed conservation of inflammatory licencing in MSCs isolated from such a diverse range of sources emphasises their overlapping biological roles and contributes to the deeper understanding of fundamental MSC biology required to optimise their therapeutic potential. While the identification of this robustly conserved secretome is important for verifying the equivalency of iPSC and tissue-derived MSCs, the lack of significant variation in licensing response between MSC populations also suggests that, though iMSCs may bypass some amount of MSC-donor variation, the impact of recipient immune responses will likely remain a source of variation in clinical outcomes. Ideally, a critical next step in developing iMSCs as a robust off-the-shelf therapy will be to investigate the efficacy of individual iMSC lines across different recipient tissue and immune cell responses.

The paired resting and licensed MSC secretomes allowed a conserved proteomic signature of MSC2 licensing to be identified, highlighting 43 proteins as key markers of this process. When analysing the biological processes that these are involved with, there was a clear distinction between the resting and licenced secretomes where resting MSC secretomes reflected a developmental, regenerative and tissue homoeostatic role, while licensed MSC secretomes reflected an immunomodulatory one. Broadly, this supports Waterman’s model of MSC polarisation, where resting MSCs provide tissue repair and regenerative functions while the MSC2 phenotype regulates immunomodulatory processes and resolves inflammation^10,15,16^. Interestingly, the licensed MSC secretome contained both pro and anti-inflammatory factors, suggesting that chemotactic factors may function to attract peripheral immune cells while immunomodulatory factors such as IDO, IL18BP and TSG6 simultaneously restrain the immune response. Inflammatory licencing also increased the concentration and complexity of the MSC secretome, with the licenced samples containing more individual proteins and at higher concentrations, likely reflecting the more complex and diverse signalling and behaviour of the MSC2 phenotype.

Despite the overall increase in complexity of the licensed MSC secretomes, there was a specific decrease in pro-regenerative proteins such collagens 1A1, 1A2 and 2A1, prostaglandin receptors and thrombospondins. This was consistent with our functional testing in which licensed, but not the resting, secretomes inhibited T cell proliferation in vitro while CM from resting MSCs more effectively induced proliferation and in vitro wound closure by fibroblasts, likely due to the increased secretion of ECM proteins. Though the concept that CM from resting MSCs is generally not immunomodulatory and will not inhibit T cell proliferation is embedded in many publications^10,11,15,28,49,77–79^, the need for inflammatory licensing of MSCs, either by cytokine stimulation, TLR agonism, or direct contact with activated immune cells, is often not explicitly described, leading to a continued lack of clarity surrounding best practices and the different roles and functions of MSC secretomes. By identifying enriched TF binding motifs across licensed MSC secretomes it was also possible to provide insights into the regulatory mechanisms involved in MSC phenotypic plasticity. The key TF networks in resting MSCs (SP3, KLF13 and SOX4) are commonly associated with the regulation of cell proliferation, differentiation and maintenance of cell identity^80–82^ and may play a role in the differentiation potential of resting MSCs, which has been reported to decrease with inflammatory licensing^83^. In contrast, the TFs driving the expression of licensed secretomes included IRF, Rel, and NF-κB family factors, all of which are known regulators of immune responses and inflammatory signalling. Specifically, IRF and NF-κB signalling drive the inflammatory response that results from TLR stimulation^56,84^, while REL, RELA and RELB form complexes with NF-κB, modulating the specificity and intensity of the resulting inflammatory response^85^. The activity of these regulatory networks implies that the switch to an MSC2 phenotype is driven by NF-κB signalling pathways, which can be activated by either inflammatory cytokines or TLR3/4 activation.

Importantly, our findings showed that the source of MSCs was responsible for more of the variability in the MSC secretome than the functional phenotype. This is of considerable interest considering the substantial differences between resting and licensed MSCs and underscores the critical importance of comprehending variability of MSC source, especially in the context of developing MSC therapies. Previously, it has not been possible to make this comparison as no prior studies have characterised the secretome of both MSC source and inflammatory phenotype in parallel. The separation of adult and natal tissue-derived MSC secretomes aligns with observations by Shin et al. who also reported that the secretome of natal tissue-derived MSCs (placenta derived and Wharton’s jelly derived-MSCs) is more diverse than that of adult tissue-derived MSCs (BM.MSCs and AT.MSCs)^45^. Kehl et al. also specifically linked the complexity of CM from Wharton’s jelly-derived MSCs to a more complete angiogenic network and higher concentrations of angiogenesis-related proteins than were identified in either BM.MSC or AT.MSC derived CM^65^. Corresponding with our findings, these suggest that some MSC lines may be more potent due simply to a greater proteomic output.

Under resting conditions, a key difference between the secretomes of iMSC/UC.MSCs and those of the adult-tissue derived MSCs was driven by secretion of proteins involved in telomerase activity and DNA/RNA metabolic processes. Enrichment of these processes emphasises the highly proliferative, and translationally active nature of the iMSCs and UC.MSCs, correlating with an observed increase in protein secretion. This may be related to in vivo MSC age, representing a more stem-like phenotype associated with ‘younger’ MSCs. Supporting this is the finding that MCAM and DSG2, which were highest in iMSC/UC.MSC secretomes, have been linked to maintenance of a stem-like phenotype^86^, while PENK, which was highest in BM.MSC/AT.MSC secretomes, has been linked to senescent or aged MSCs^87–89^. Confirming that these changes have functional effects, CM from iMSCs and UC.MSCs, which displayed a ‘younger’ profile, increased both the proliferation and migration of hDFs to a greater extent than those derived from adult tissue sources. BM.MSC/AT.MSC secretomes were characterised by the upregulation of proteins linked to ECM structure and organisation and proteolytic processes, suggesting active involvement in tissue repair and microenvironment maintenance and perhaps suggesting their advantage for tissue-forming applications, although increased secretion of ECM proteins may also contribute to fibrotic signalling pathways.

Telomerase activity and DNA/RNA biosynthetic processes remained a key difference between iMSC/UC.MSC and adult tissue-derived secretomes after inflammatory licencing. Despite the broad upregulation of immunomodulatory proteins in licensed secretomes, some differences in the levels of specific immunomodulatory proteins were observed. Licensed UC.MSC secretomes contained different concentrations of several chemotactic factors, suggesting that they may be comparatively more chemotactic towards neutrophils than other MSCs, but less chemotactic towards lymphocytes and monocytes. While the chemotactic effect of UC.MSCs has not been directly compared with that of MSCs from other sources, general differences in the mechanisms by which they exert their immunomodulatory effects has been described. Song et al. reported an increase in IL-6 and prostaglandin E2, in UC.MSC secretomes in comparison to BM.MSC secretomes. Despite this they also reported that UC.MSCs were able to functionally exert comparable immunomodulatory effects^90^. Overall, the variations in MSC secretory profiles mirror distinct physiologies of the MSC source, with iMSCs and UC.MSCs producing a secretome that reflects natal tissue origins and the effective regression of in vivo age that occurs with iPSC-based technologies^91,92^, while older, adult tissue-derived MSC secretomes reflect tissue homoeostatic and fibrotic phenotypes.

Ultimately, the differences in MSC secretome are important due to the functional effects that they exert. Importantly, experimental validation confirmed that the differences observed between resting and licensed MSC secretomes were predictive of the target cell response, in models of wound closure, angiogenesis, macrophage polarisation and T cell proliferation. These assays showed that only licensed MSC CM could induce significant polarisation of macrophages or inhibition of T cell proliferation, while the resting MSC CM better supported wound closure and angiogenic processes. These functional data support expectations from analysis of the CM composition that immunomodulatory properties are present only after inflammatory licensing and that the resting secretome more effectively promotes pro-regenerative and wound healing processes^10,11,15,50^. Mechanistically the role of many individual factors driving the functional effects of the MSC secretomes are well established (e.g. IDO, PDGE2, TSG6, VEGF). The comprehensive profiling in this dataset can provide new information, by facilitating a parallel comparison of these factors between different donors, sources and biological phenotypes; and context, with a complete proteomic profiles including both recognised and novel targets and highlighting unique expressional patterns between and within MSC populations.

Overall, this study presented a detailed atlas of resting and MSC2-licensed MSC secretomes across multiple tissue sources, donors and iPSC-platforms. Importantly, we demonstrate for the first time that immunomodulatory plasticity and the MSC2 phenotype are recapitulated by iMSCs which supports their equivalency to tissue-derived MSCs in a process which is critical for their therapeutic potential. The data also identify a conserved profile of MSC2 inflammatory licensing in which resting MSCs produce a pro-regenerative secretome which rapids switches to an immunomodulatory profile upon inflammatory stimulation. In tandem, we describe the variations between MSC secretory profiles from different sources, where iMSC secretomes were most similar to UC.MSC secretomes, and AT.MSC and BM.MSC secretomes were most similar to each other. This reflects distinct physiologies of MSC sources, with strong emphasis on in vivo age and provides valuable fundamental insights into MSC heterogeneity. Collectively, this comprehensive data on the composition of MSC secretome provides insights into the molecular mechanisms underlying the therapeutic effects of MSCs and the differences by source and functional state. This can be used to inform the design of more effective MSC-based therapies by identifying the most suitable MSC source for a particular application, allowing the development of tailored culture conditions and/or preconditioning methods to enhance the therapeutic potential of these cells, or even identifying specific factors that can be adapted for pharmaceutical intervention.

## Methods

### Derivation of the MSC working panel

A working panel of MSC populations was created, with 13 human MSC populations, including three donors of bone marrow, adipose tissue and umbilical cord,-derived MSCs, three independent batches of Cynata Therapeutics’ clinical iMSC product, CYP001 and one batch of commercial-grade iMSCs, purchased from Cellular Dynamics Incorporated (CDI). We have complied with all relevant ethical regulations including the Declaration of Helsinki.

#### Bone marrow-derived MSCs

Three donors of bone marrow-derived MSCs, donor IDs 18TL113327 (BM.MSC 1), 0000684888 (BM.MSC 2) and 0000539540 (BM.MSC 3), were purchased at P2 (Lonza; #PT-2501). P2 vials were thawed as per manufacturer’s instructions and plated in a T175 flask in MSC basal medium consisting of DMEM with 10% foetal bovine serum (FBS).

#### Adipose tissue-derived MSCs

Human adipose tissue samples were taken from the lower abdomen of patients undergoing routine C-section with informed consent (Ritchie Centre Human TissueBank, Hudson Institute of Medical Research, HREC reference 12387B). Tissue was kept at RT and processed within four h of collection. Briefly, the tissue was washed using DPBS (ThermoFisher; #14040216) supplemented with 1× Antibiotic-Antimycotic containing 100 units/mL of penicillin, 100 µg/mL of streptomycin and 0.25 µg/mL of Gibco Amphotericin B (AA) (ThermoFisher; #15240062), mechanically dissociated using scalpel blades and collected in a 50 mL falcon tube. Dissociated tissue was digested in 2 mg/mL collagenase type I (Life Technologies; #A1064401) in DMEM) at 37 °C for 1 h under gentle agitation. Digested samples were diluted 1:1 with MSC high glucose basal medium, sequentially filtered through 100 µm and 40 µm filters, and centrifuged to pellet the cells. This pellet was treated with 1:3 mixture of DMEM: erythrocyte lysis buffer (155 mM NH4Cl (ThermoFisher; #254134), 10 mM KHCO3 (Fisher Scientific; #P235-500), 0.1 mM ethylenediaminetetraacetic acid (EDTA) (ThermoFisher; #15575020) for 5 min. at RT. The remaining cells were resuspended in DMEM-HG (Gibco; #11965092) with 1% AA and 20% FBS (Scientifix; #SFBS-AU) and plated at 2.5 × 10^3^ cells/cm^2^. Media was replaced after 24 h When SVF reached 70% confluence cells were detached using TrypLE (ThermoFisher; #12605010) for 3 min. at 37 °C and replated at 2.5 × 10^3^ cells/cm^2^ at P0 in MSC basal media.

#### Umbilical cord-derived MSCs

Human umbilical cord tissue samples were collected with informed consent (Ritchie Centre Human TissueBank, Hudson Institute of Medical Research, HREC reference 12387B). Tissue was kept at RT and processed within 4 h. of collection. Briefly, the tissue was washed using DPBS (ThermoFisher; #14040216) supplemented with 1× AA (ThermoFisher; #15240062). Umbilical vein and arteries were removed using scalpel blades and the remaining Wharton’s jelly and cord tissue was sectioned into 5 mm^2^ explants. Explants were plated 1 per 5 cm^2^ in a cell culture dish in DMEM-HG (Gibco; #11965092) with 1% AA and 20% FBS (Scientifix; #SFBS-AU). Media was replaced after 24 h. When outgrowth was visible (7–10 days) explants were removed and adhered cells were detached using TrypLE (ThermoFisher; #12605010) for 3 min. at 37 °C and replated at 2.5 × 10^3^ cells/cm^2^ at P0 in MSC basal media.

#### iPSC-derived MSCs

To cover the variability inherent in iMSC production and ensure the relevance of our findings to clinical practice, three batches (CYN002, CYN004 and CYN005) of Cynata therapeutics’ clinical-grade iMSC product CYP001^TM^ were provided by Cynata Therapeutics’. P2 vials were thawed as per provider’s recommendations and plated in a T175 flask in MSC basal medium. This selection encompassed the entire range of iMSCs under investigation in clinical trials at the time, while the availability and verified of these iMSC lines ensured that our study’s conclusions are broadly applicable.

An additional batch of commercial-grade iMSCs (iCell Mesenchymal Stem Cells^TM^) were purchased from CDI USA (Cellular Dynamics; #R1098). P2 vials were thawed as per the manufacturer’s recommendations and plated in a T175 flask in MSC basal medium.

### MSC maintenance

MSC lines were cultured in MSC basal media at 37 °C and 5% CO_2_. All cultures were tested and confirmed free of mycoplasma every 3 months using a Mycoalert kit (LT07-118; Lonza). When cultures reached 80% confluence cells were detached using TrypleE Express Enzyme (ThermoFisher; #12604013) and passaged at a 1:4 ratio. All experiments were performed using passage six MSC lines and prior to all experiments, cells were serum-starved for 12 h in DMEM containing 0.5% FBS.

### MSC characterisation and licensing

Multilineage differentiation of MSC lines was assessed by differentiation into osteocytes, adipocytes and chondroblasts as previously described^93^. After 21 days differentiated cell cultures were fixed and stained using (i) Alizarin Red (Sigma Aldrich; #A5533), (ii) Oil Red O (Sigma Aldrich: #O0625), or (iii) Alcian blue (Sigma Aldrich; #A5268).

MSCs were characterise on surface markers recommended but the ISCT minimal criteria^3^ CD14, CD19, CD34, CD45, CD73, CD90, CD105 and HLA-DR by flow cytometry. 5 × 10^5^ MSCs were co-stained for 1 h at room temperature according to standard procedures with antibodies listed below. Thirty thousand events were acquired using LSR Fortessa X20 (BD Bioscience, USA) and data sets were analysed using FlowJo software (Tree Star, Inc., UCA).

MSC lines were plated in complete MSC basal media and allowed to adhere overnight before licensing. Licencing was performed by culturing MSCs in DMEM-LG supplemented with 10% heat inactivated FBS (HI-FBS, 58 °C for 30 min) 15 ng/ mL hIFN-*γ* (Peprotech; #300-02) and 15 ng/mL hTNF-*α* (Peprotech; #300-01 A) for 48 h. Resting MSCs were cultured in parallel in DMEM-LG supplemented with 10% heat inactivated FBS MSC.

Licensed phenotype was confirmed by flow cytometry analysis and quantification of IDO in conditioned media. After 48 h culture with or without hIFN-γ/ hTNF-*α* exposure, 3 × 10^5^ MSCs were co-stained for HLA-DR and HLA-ABC for 1 h at room temperature according to standard procedures. Antibodies and concentrations are listed below. Thirty thousand events were acquired using LSR Fortessa X20 (BD Bioscience, USA) and data sets were analysed using FlowJo software (Tree Star, Inc., UCA).

Concentration of secreted IDO was assessed using human Indoleamine 2,3-dioxygenase IDO DuoSet enzyme-linked immunosorbent assay (ELISA) (RD systems; #DY6030B-05), as per the manufacturer’s instructions.

### MSC CM production and harvest

MSC lines were plated in complete MSC basal media and allowed to adhere overnight before MSC licensing. After 48 h the medium was aspirated and cells were washed gently three times in excess PBS. Conditioning medium (1 mL/1 × 10^5^ cells), consisting of DMEM-LG, no phenol red (Gibco; #31053028) supplemented with 1:100 ITS (Sigma-Aldrich; # I3146-5ML) was added for a further 48 h before the conditioned media (CM) was collected and filtered using a 0.2 µm filter to remove cell debris. CM was processed for LC-MS/MS immediately or aliquots stored at -80 °C for further analyses.

### QC and proteomic profiling of MSC secretomes

Liquid chromatography-tandem mass spectroscopy (LC-MS/MS) experiments were performed by Monash Proteomics and Metabolomic Facility. Proteins were retrieved from 50 µl of CM using 5 µl Strata Clean resin beads per mL of CM (Agilent Technologies, USA) and further processed for in-gel digestion. 5 µl peptide extract was analysed by LC-MS/MS on an EASY-nLC1000 chromatograph connected to a QExactive HF mass spectrometer (ThermoFisher Scientific) using a Pepmap100 Trap C18 300 mm × 5 mm (Thermo Fisher Scientific) and a C18 separation column (3 µm, 100˚A, 75 µm × 15 cm, Nikkyo Technos, Tokyo, Japan) by applying a 120 min. gradient.

LC-MS/MS data was processed with MaxQuant (version 1.5.4.1) using default settings for peak detection, strict trypsin cleavage rule allowing for up to three missed cleavages, variable oxidation on methionine, deamidation of asparagine and glutamine and acetylation of protein N-termini with strict carbamidomethylation of cysteines. Match between runs was used within each sample group with a retention time window of 1 min. Label free quantification (LFQ) was performed using classical normalisation with LFQ separated into parameter groups. The fragment spectra were interpreted with the UniProt Homo sapiens database (UP000005640) accepting only protein identifications with at least two razor peptides at a 1% false discovery rate (FDR) cutoff.

### Processing, analysis and visualisation of LC-MS/MS data

For LC-MS/MS data analysis, protein lists retrieved from MaxQuant pipeline were processed using LFQ Analyst^94^, removing contaminant proteins, reverse sequences, proteins identified ‘only by site’ and those detected in the unconditioned media control (DMEM-LG + 1:100 ITS). Remaining protein lists were mapped to Gene Ontology term: extracellular space (GO:0005615) (GSEA database https://www.gsea-msigdb.org/gsea/msigdb/human/genesets.jsp?collection=GO:CC). to filter for secreted protein fractions. Where a protein was positively identified in two of three samples within a group the missing value was imputed as the median transformed intensity. Where proteins had at least one positive identification across all samples missing values were imputed from the low end of the Log2 transformed intensity distribution from each individual sample using Perseus (version 1.5.5.3) as suggested by Lazar et al.^95^. Data visualisation, DE and enrichment analysis were performed using iDEP 1.1^96^ (http://bioinformatics.sdstate.edu/idep96/). K-means clustering was done using averge linkage, using the elbow methodology to determine optimal number of clusters. Limma package was used for identification of differential expressed DE proteins between comparison pairs with an FDR cut off of 0.05 and a fold change ≥2.

### Human fibroblast proliferation and migration assays

To assess the effect of resting and licensed CM on human dermal fibroblast (hDF) proliferation hDFs were plated at 2 × 10^4^ cell/cm^2^ and allowed to adhere overnight. Media was exchanged for CM supplemented with 0.5% HI FBS and hDF proliferation was assessed at days 1, 3, 5 and 7 using CellTiter 96® AQueous One Solution Cell Proliferation Assay (MTS) (Promega; #G3582) as per the manufacturer’s instructions.

To assess the migration of hDFs, an in vitro monolayer wound assay was conducted. hDFs were seeded in 48-well plates in DMEM supplemented with 10% FBS until they reached confluence. Subsequently, a wound was made across the cultured cells using a 20 µl pipette tip. The culture medium was immediately replaced with CM. Cell migration was evaluated using time-lapse microscopy every 6 h. Wound closure was quantified by calculating the area under the curve (AUC) of wound closure over 72 h. AUC measures the total extent of wound healing over time, capturing both the rate and completeness of healing and providing a more comprehensive assessment of wound healing dynamics which account for variations in healing speed and the ultimate closure outcome.

### Angiogenic tube formation assays

To assess the effect of resting and licensed CM on angiogenic tube formation, human umbilical vein endothelial cells were plated directly into resting or licensed CM at 5 × 10^4^ cells per well on matrigel coated 3D 96-well plates (Ibidi; #89646). Angiogenic tube formation and collapse was recorded ever hour over 48 h. using a MuviCyte live-cell imaging system at 37 °C and 5% CO_2_. Time course images were analysed using ImageJ AngiogenicAnalyser plugin^97^ to calculate mesh index, number of nodes, number of branches. and branching length.

### Macrophage polarisation assays

To assess the effect of resting and licensed CM on polarisation of macrophages, THP-1 cells were treated with 5 ng/mL phorbol-12-myristate 13-acetate (PMA) for 24 h. after which the culture media was exchanged for PMA-free RPMI for a further 24 h. At 48 h. the remaining non-adherent cells were removed and media was exchanged for either resting or licensed MSC CM for an additional 48 h. Control wells were treated with either IFNγ (20 ng/mL) + LPS (200 ng/mL) for M1 or IL-4 (20 ng/mL) + IL-13 (20 ng/mL) for M2. Polarisation of macrophages was assessed by counter staining CD80 (BDbiosciences; #567339) and CD206 (BDbiosciences; #555953) surface markers or by flowcytometry.

### T-cell proliferation assays

To assess the ability of MSC CM to inhibit T-cell proliferation murine pan T-cells were isolated using Miltinye Pan T-cell Isolation Kits (Miltenyi Biotech; #130-095-130) as per the manufacturer’s instructions. T-cells were plated at 2 × 10^4^ cells per well in a 96-well U-bottom plate resuspended in DMEM-HG or CM with or without 5 ng/mL Phytohaemagglutinin (PHA) (ThermoFisher; #00-4977-93). T-cells were cultured for 5 days at 37 °C and 5% CO_2_ before quantification of cell number using MTS reagent (Promega; #G3582) as per the manufacturer’s instructions.

### Statistical analysis

Minimum *n* = 3 technical replicates for all analyses. Averages are presented with respective individual data points. Comparisons between data sets with more than two means was performed by One-way anova with Tukey/Bonferroni post hoc multiple comparisons. *P* < 0.05 was considered significant.

## Supplementary information

Supplementary Information

Supplementary Data 1

## Data Availability

Proteomics data generated for this study have been deposited in ProteomeXchange database (PXD055113). Source data are available with the paper and all other data or materials are available from the corresponding author upon reasonable request.
