# Supplementary material for: Proteomic profiling of iPSC and tissue-derived MSC secretomes reveal a global signature of inflammatory licensing
Source: NPJ Regen Med. 2025 Feb 4;10:7. doi: 10.1038/s41536-024-00382-y (PMC11794695; doi:10.1038/s41536-024-00382-y)
Supplement: Supplementary file 1 — Supplementary Information [file 41536_2024_382_MOESM1_ESM.pdf]

Supplementary Information

Supplementary Table 1: Details of MSC lines.

Population name	Source	Source ID	Age	Sex
**CYN.iMSC 1**	Cynata Therapeutics'	CYP002	NA	NA
**CYN.iMSC 2**	Cynata Therapeutics'	CYP003	NA	NA
**CYN.iMSC 3**	Cynata Therapeutics'	CYP005	NA	NA
**CDI.iMSC 4**	Cellular Dynamics Inc.	MSC-301-010-001-PT	NA	NA
**BM.MSC 1**	Lonza Bioscience	3327	31y	M
**BM.MSC 2**	Lonza Bioscience	4888	33y	M
**BM.MSC 3**	Lonza Bioscience	9450	48y	F
**UC.MSC 1**	Tissue donation	NA	0	F
**UC.MSC 2**	Tissue donation	NA	0	M
**UC.MSC 3**	Tissue donation	NA	0	F
**AT.MSC 1**	Tissue donation	NA	29y	F
**AT.MSC 2**	Tissue donation	NA	30y	F
**AT.MSC 3**	Tissue donation	NA	29y	F

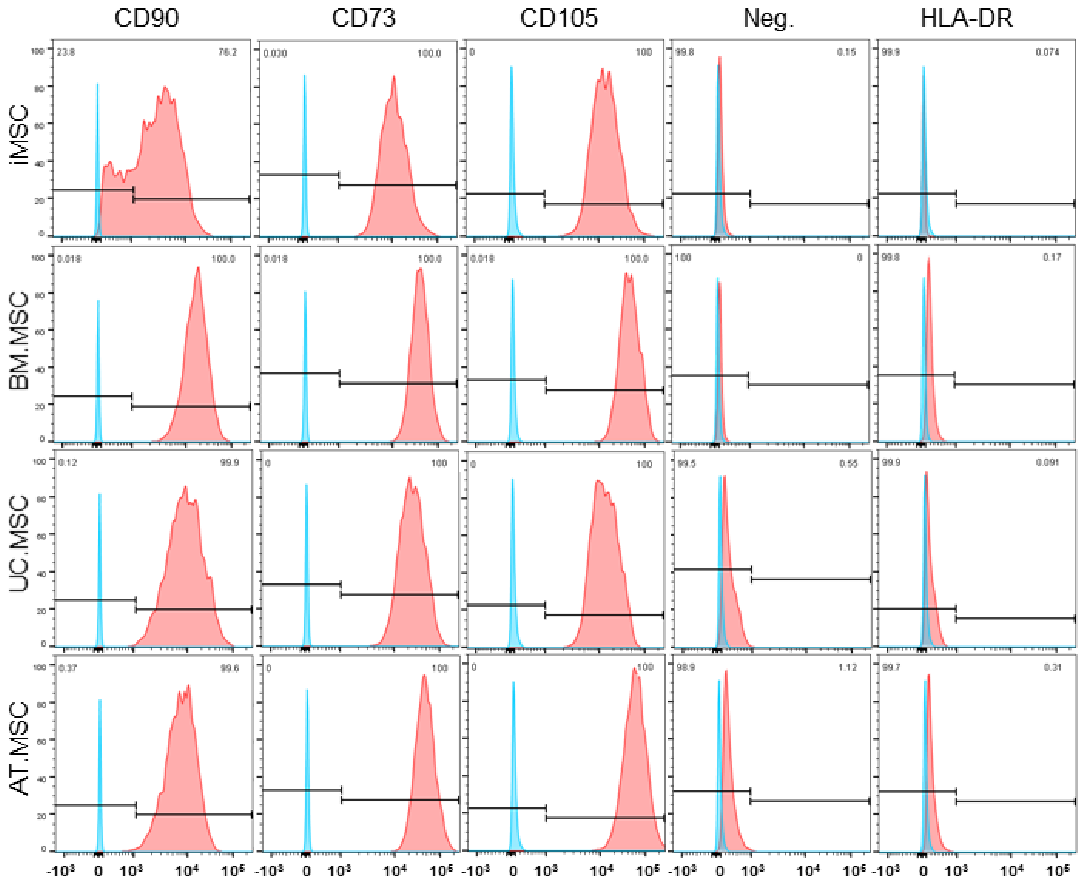

Supplementary Figure 1: Expression of ISCT minimal criteria markers across MSC lines. Flow cytometry shift histograms of representative MSC lines (pink) co-stained for ISCT minimal criteria markers, CD73, CD90, CD105, Negative cocktail of CD34, CD45, CD14, and CD19, and HLA-DR. MSC populations were gated against an isotype control (blue). Percentage of positive gate (MSC line) is shown on the top right. Statistics were performed using FlowJo V10. All MSC lines adhered to the ISCT minimal criteria. Histograms are representative of each MSC source.

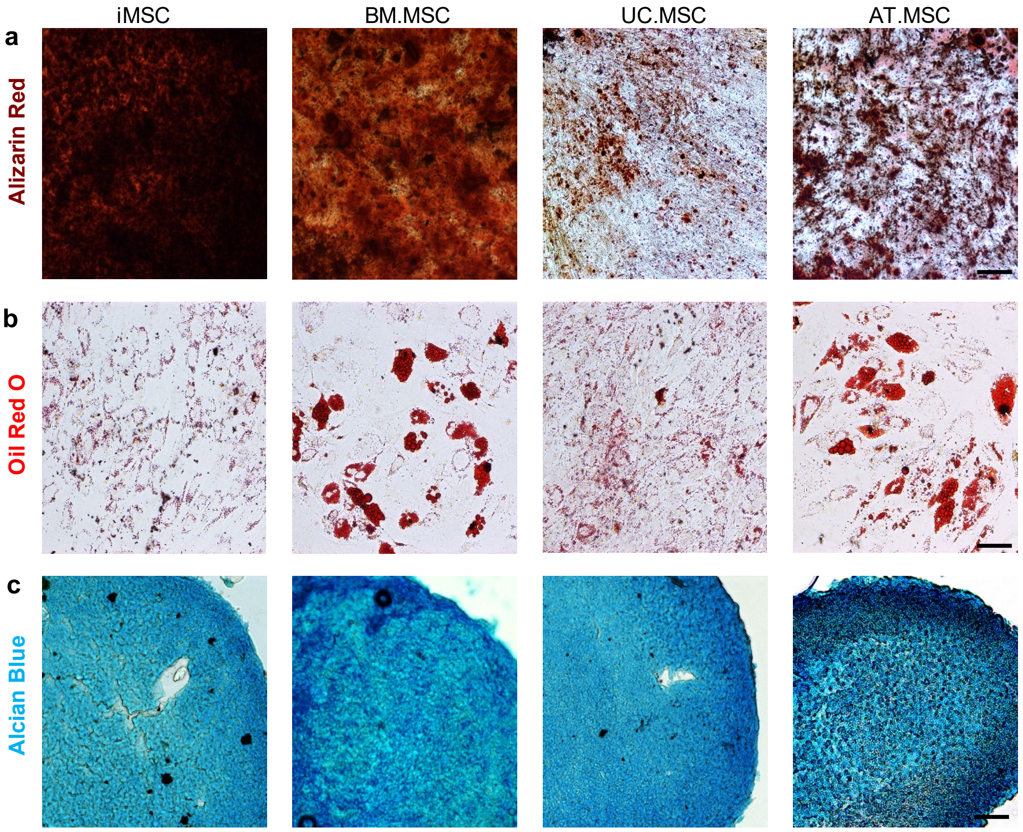

Supplementary Figure 2: Trilineage differentiation potential of MSC lines. (a) Osteogenic MSC cultures stained for extracellular mineral depositions using Alizarin red (red), scale bar is 200nm. **(b)** Adipogenic MSC cultures stained for intracellular lipid deposits using Oil Red O (red), scale bar is 200nm. **(c)** Chondrogenic MSC pellet cultures stained for glycosaminoglycans using Alcian blue (blue), scale bar is 50nm. All differentiations were performed in triplicate across all 13 MSC lines. Images are representative of each MSC source.

a

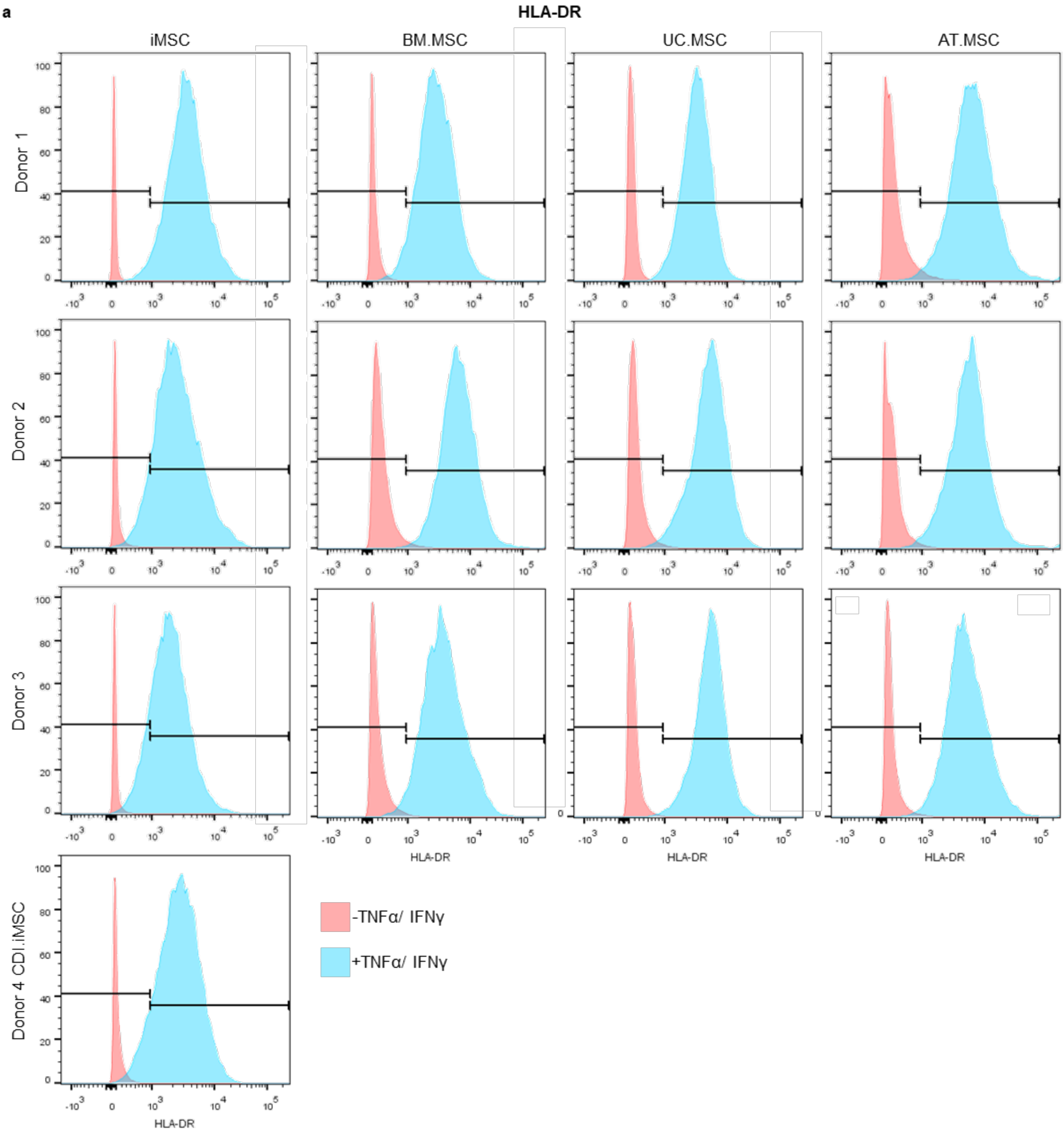
HLA-DR
iMSC
BM.MSC
UC.MSC
AT.MSC
Donor 1
Donor 2
Donor 3
Donor 4 CDI.iMSC
HLA-DR
-TNFα/ IFNγ
+TNFα/ IFNγ

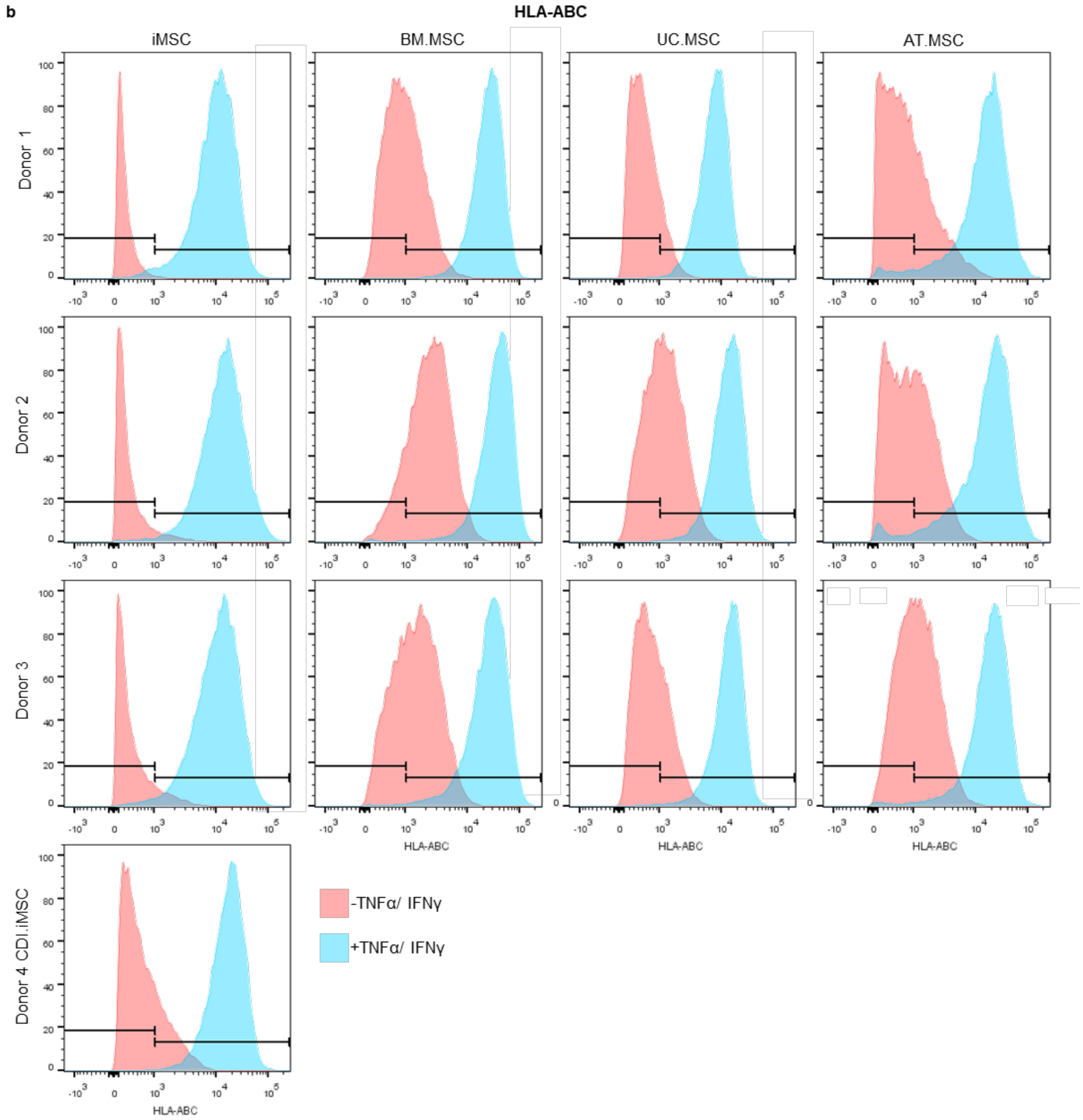

Supplementary Figure 3: Expression of inflammatory licensing markers HLA-ABC and HLA-DR across 13 MSC lines with and without inflammatory cytokines. Flow cytometry shift histograms of 13 MSC lines costained for HLA-DR **(a)** and HLA-ABC **(b)** with (blue) and without (pink) 48 h. exposure to inflammatory cytokines. MSC populations were gated against an isotype control. Statistics were performed using FlowJo V10.

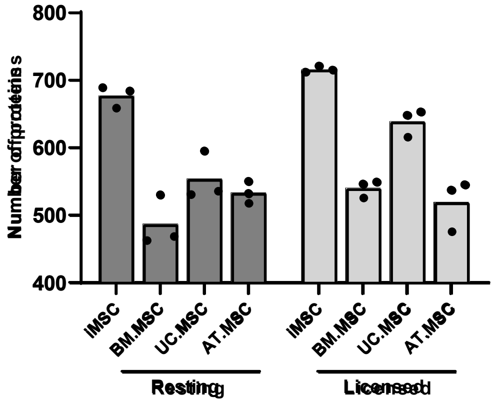

Supplementary Figure 4. Secreted protein number by MSC source. Bar charts shows number of secreted proteins detected in each MSC source under resting and licensed conditions. Bars show mean number of detected proteins with individual points for the number of proteins detected in CM from each MSC line.

Supplementary Table 2. Fold change, adjusted P values, and average intensity across MSC source of proteins significantly differentially expressed between resting and licensed MSC secretomes.

Protein	LICENSED-RESTING log2FC	LICENSED-RESTING adjPval	Resting iMSC average	Resting BM.MSC average	Resting UC.MSC average	Resting AT.MSC average	Licensed iMSC average	Licensed BM.MSC average	Licensed UC.MSC average	Licensed AT.MSC average
CXCL10	25.20518	3.74E-27	0	0	0	0	25.19633	25.51879	24.73365	25.37221
CCL5	24.30675	0.000107	0	0	5.868606	0	24.03559	26.30138	26.26406	26.50182
CXCL9	23.20099	0.000289	0	0	6.087522	0	24.19218	24.92397	24.74814	25.00082
CXCL11	23.16887	2.81E-26	0	0	0	0	23.66524	22.80353	23.61039	22.58309
IL18BP	22.20908	8.91E-25	0	0	0	0	21.0827	22.67429	22.5579	22.55141
CTSS	21.81849	2.81E-26	0	0	0	0	20.94779	22.75214	21.3063	22.2909
IL4I1	19.21653	1.31E-21	0	0	0	0	18.34988	20.34178	18.74171	19.45583
SECTM1	18.90335	0.00193	4.242349	0	0	0	19.41656	20.42156	20.17663	19.94032
ALDOB	18.45965	0.001704	4.031933	0	0	0	20.12016	19.60679	18.98642	19.22032
HLA-B	17.05918	0.110052	13.54788	0	6.137445	0	23.7039	22.11589	21.72343	20.56264
ICAM1	16.48594	0.001767	9.041057	0	15.02343	0	22.93595	21.79478	23.34803	21.9985
CXCL16	15.78782	0.181996	0	5.751015	0	0	9.04214	21.21865	19.63379	19.18733
SOD2	15.39521	0.158168	0	0	5.684843	0	20.91829	6.5069	20.96564	18.72779
ISG15	15.19771	0.034085	19.84673	0	0	5.317846	23.7856	20.54915	21.52045	20.39998
KLK10	15.00834	0.045193	0	0	0	0	4.253965	18.6399	18.00609	19.41976
RPL27	14.89631	0.134233	0	0	5.842143	5.866747	19.08307	17.46091	17.29555	17.34312
SLIT2	14.73334	0.253267	3.193779	4.640242	5.523147	4.815393	18.43369	20.35829	19.46169	18.83877
C4B	14.66862	0.067694	4.464727	0	17.54601	0	17.06686	21.70782	21.12913	20.83644
SCRG1	-14.0619	0.045503	18.46373	19.38281	20.55713	19.33675	3.842075	0	17.66552	0
SERPINB1	14.05762	0.141	11.50455	5.30929	0	0	19.87557	17.3589	19.28232	16.67893
C2	14.0262	0.232806	0	0	0	5.789147	4.433212	19.19289	18.31683	20.20641
SDCBP	-13.084	0.048762	19.54046	18.44	18.77165	17.76668	16.72074	0	5.188903	0
CTSC	12.73066	0.102224	4.002463	0	0	0	18.19209	0	17.38733	19.30684
APOC3	12.71503	0.134233	0	5.954646	0	0	0	18.61656	18.54673	19.99003
DYNC1H1	12.58995	0.001479	18.02834	0	0	0	20.08948	15.50084	18.1064	14.97174
GBP1	12.09092	0.074168	16.46272	18.38598	4.741158	0	24.38719	20.14689	22.94127	20.58911
IFI30	11.84489	0.135496	3.838269	18.49869	0	18.87118	20.95537	22.97829	21.89751	22.61614
LAMA2	-11.7756	0.333271	11.89878	18.55593	21.17003	19.57275	0	0	19.12225	4.976296
LAP3	11.74466	0.232778	17.52748	4.647383	5.250506	15.74167	24.71737	21.33639	22.77862	21.43457
CXCL8	11.5305	0.730611	4.423615	0	21.88431	6.299521	21.38435	20.81592	22.63619	13.7484
GBA	11.35736	0.645018	15.47277	7.035858	21.58377	0	21.2437	22.51115	22.95021	22.9655
SLC1A5	-11.2773	0.241709	19.08649	17.78675	20.71443	0	12.35721	0	0	0
MDK	11.25577	0.203486	21.68342	5.547816	20.88467	0	23.66279	23.1888	23.87355	22.66085
CX3CL1	11.08015	0.345178	0	0	0	0	5.073306	20.09618	0	19.31104
RPL30	10.94454	0.298851	18.78828	0	0	0	20.29045	6.216413	18.17573	18.13528
RARRES1	10.52572	0.244446	0	5.35327	19.06783	15.84517	13.5667	20.36673	24.07015	24.2846
STAT1	10.46891	0.141181	14.98114	4.6527	0	14.62189	22.48128	17.31812	20.09436	16.31664
MYL3	10.24886	0.719052	21.18777	6.720099	0	0	21.77945	20.23016	20.5497	6.601127
XYLT1	-10.0687	0.7136	12.11023	18.20129	19.57627	17.47983	3.934206	5.594218	17.51383	0
MFAP4	-9.77495	0.030088	20.22215	17.58517	18.51408	18.289	0	0	17.63577	18.15301
PSMB9	9.773744	0.048762	0	0	0	0	20.66449	0	18.14051	0
HNRNPK	9.61363	0.372231	20.781	0	0	0	20.79662	5.360002	17.52323	15.81121
PSMB8	9.557877	0.047207	0	0	0	0	20.06381	0	17.88797	0

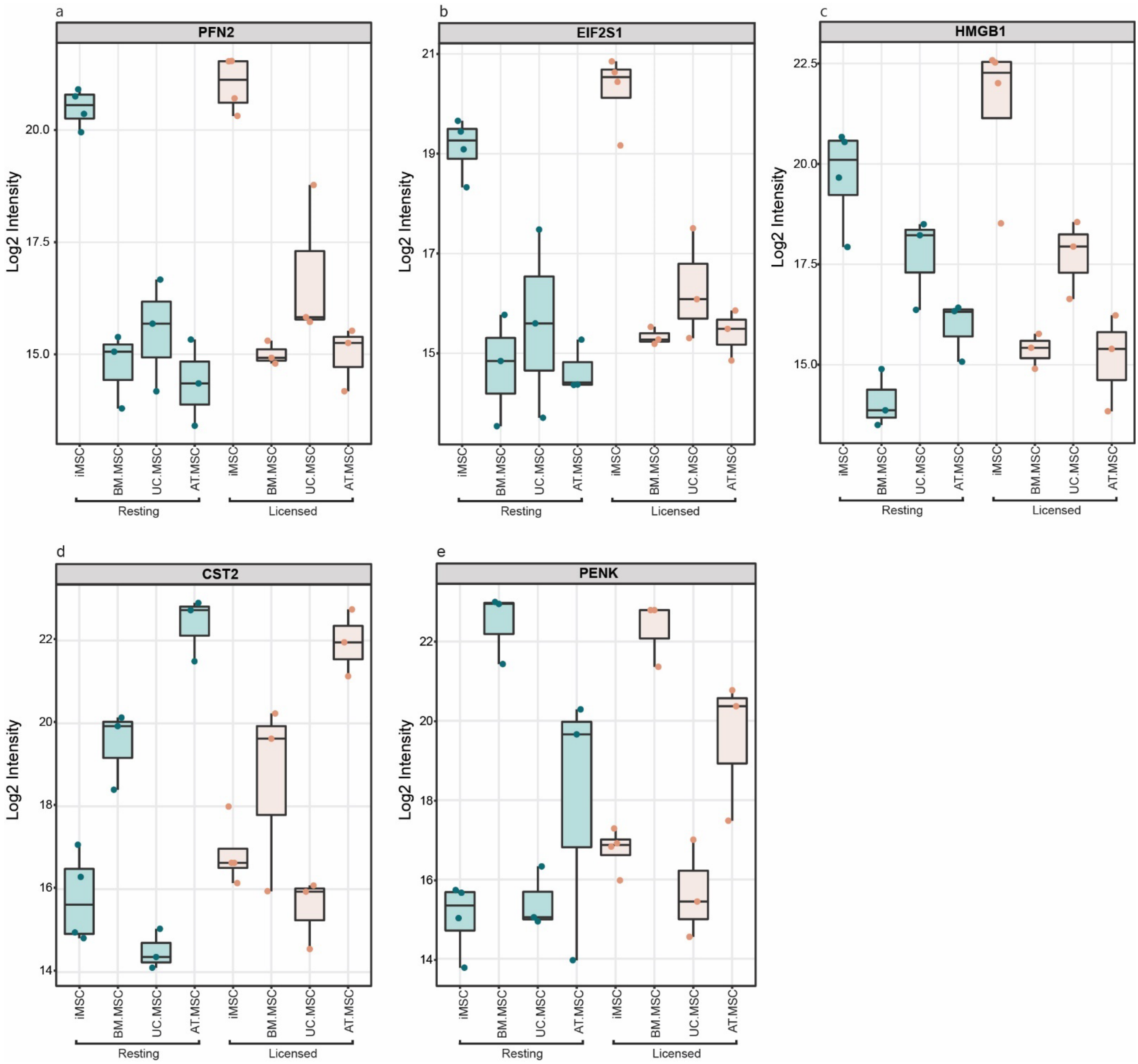

Supplementary Figure 5. Intensity of loading proteins across MSCs from different sources under either resting or licensed conditions. Box plots show mean and quartiles of $\log_2$ protein intensity for **(a)** PFN2, **(b)** EIF2S1, **(c)** HMGB2, **(d)** CST2, and **(e)** PENK across MSCs from different sources, under resting (teal) and licensed (coral) conditions with individual points for the intensity of proteins CM from each MSC line.

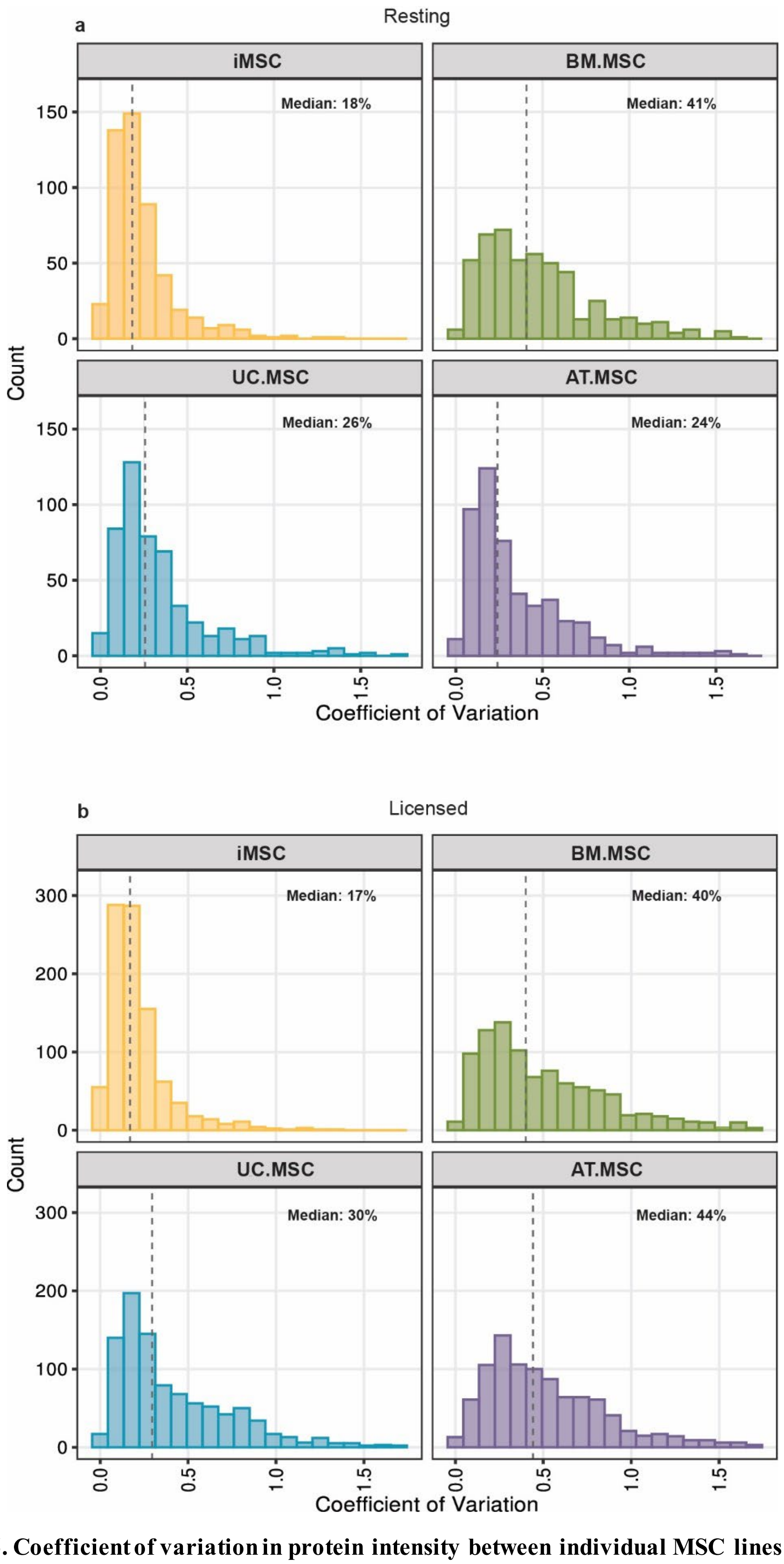

Supplementary Figure 6. Coefficient of variation in protein intensity between individual MSC lines from each source. Frequency histograms show the number of proteins (y) across binned variance coefficients (x). MSCs are separated by source under both **(a)** resting and **(b)** licensed conditions. The median coefficient of variation is displayed in the upper right. Plots were generated using LFQ Analyst.

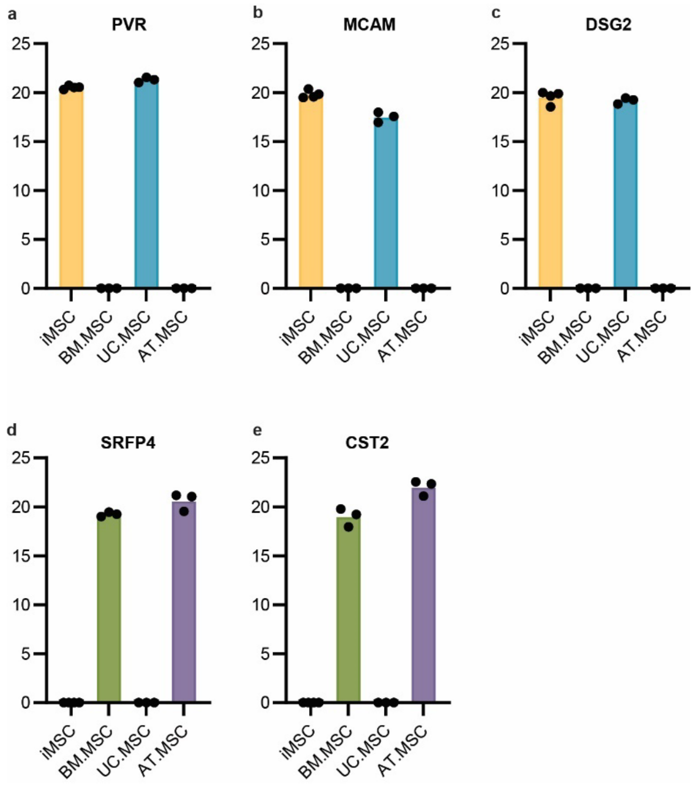

Supplementary Figure 7. Intensity of PC1 loading proteins across resting MSCs. Bar plots show mean log_2 protein intensity for **(a)** PVR, **(b)** MCAM, **(c)** DSG2, **(d)** SRFP4, and **(e)** CST2 with individual points indicating the intensity of proteins CM from each MSC line.

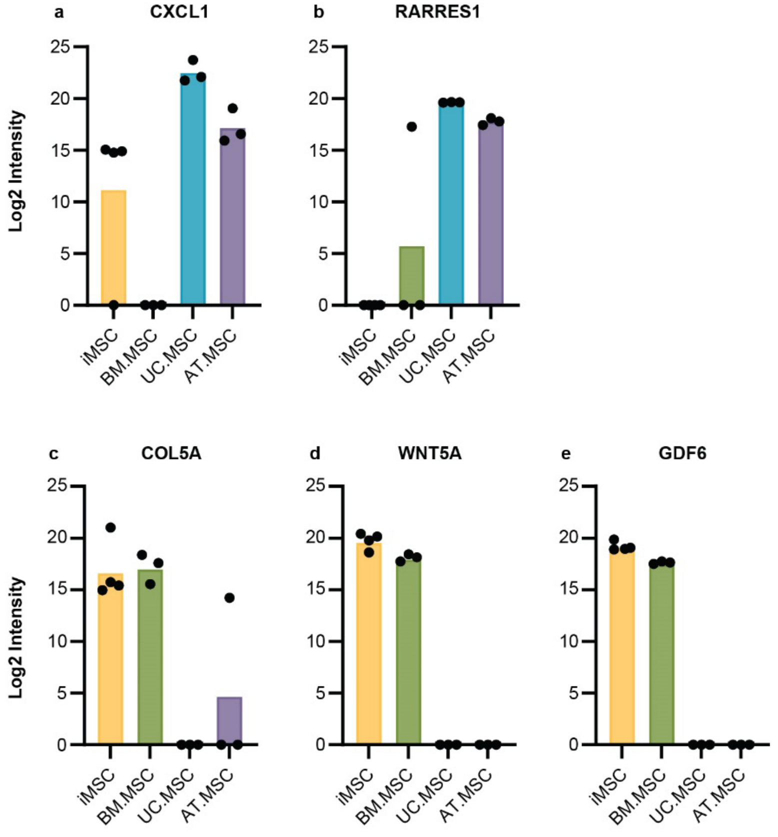

Supplementary Figure 8. Intensity of PC2 loading proteins across resting MSCs. Bar plots show mean log_2 protein intensity for **(a)** CXCL1, **(b)** RARRES1, **(c)** COL5A, **(d)** WNT5A, and **(e)** GDF6 with individual points indicating the intensity of proteins CM from each MSC line.

Supplementary Table 3. Enriched processes between resting MSC secretomes. Generated using iDEP 1.1[96]

	Pathway	FDR	Fold Enrichment
**Cluster 1**	Negative regulation of peptidase activity	6.74E-03	13.59014
	Extracellular matrix organization	1.03E-02	10.87211
	Extracellular structure organization	1.03E-02	10.84157
	External encapsulating structure organization	1.03E-02	10.75097
	Negative regulation of proteolysis	1.17E-02	9.947423
	Negative regulation of hydrolase activity	1.17E-02	9.649
	Regulation of peptidase activity	2.60E-02	7.582711
	Regulation of proteolysis	1.03E-02	6.502335
	Proteolysis	6.58E-03	4.323381
	Tissue development	6.74E-03	4.070527
**Cluster 2**	Positive regulation of establishment of protein localization to telomere	6.85E-08	62.25161
	Regulation of establishment of protein localization to telomere	1.09E-07	56.59238
	Regulation of protein localization to Cajal body	1.09E-07	56.59238
	Positive regulation of protein localization to Cajal body	1.09E-07	56.59238
	Regulation of establishment of protein localization to chromosome	1.70E-07	51.87634
	Protein localization to nuclear body	1.70E-07	51.87634
	Protein localization to Cajal body	1.70E-07	51.87634
	Positive regulation of protein localization to chromosome telomeric region	2.73E-07	47.88586
	Protein localization to nucleoplasm	4.46E-07	44.46544
	Regulation of protein localization to chromosome telomeric region	6.97E-07	41.50108
**Cluster 3**	Negative regulation of mRNA metabolic process	8.81E-04	22.81087
	Regulation of mRNA splicing via spliceosome	1.99E-03	18.80897
	Regulation of DNA biosynthetic process	3.02E-03	16.49402
	Regulation of mRNA processing	3.53E-03	14.68645
	BMP signaling pathway	4.35E-03	12.68771
	Response to BMP	4.97E-03	11.91235
	Cellular response to BMP stimulus	4.97E-03	11.91235
	Regulation of RNA splicing	5.81E-03	11.10996
	Morphogenesis of a branching epithelium	6.17E-03	10.82941
	Regulation of mRNA metabolic process	4.64E-04	10.62153
**Cluster 4**	Protein folding in endoplasmic reticulum	1.17E-06	58.26691
	Extracellular matrix assembly	1.43E-08	25.685
	Collagen fibril organization	7.55E-12	23.30676
	Basement membrane organization	1.05E-05	22.67685
	Embryo implantation	6.15E-06	17.79789
	Positive regulation of axon extension	2.49E-04	17.48007
	Regulation of extracellular matrix organization	1.05E-05	16.31473
	Transforming growth factor beta production	4.01E-04	15.53784
	Negative regulation of coagulation	8.54E-05	15.25534
	Collagen biosynthetic process	4.30E-04	15.20006
**Cluster 5**	Viral entry into host cell	4.60E-03	13.51969
	Entry into host	4.63E-03	13.02159
	Extracellular matrix organization	1.79E-05	12.54475
	Extracellular structure organization	1.79E-05	12.50951
	External encapsulating structure organization	1.79E-05	12.40497
	Movement in host environment	5.34E-03	11.29727
	Heart morphogenesis	2.81E-03	11.28868
	Cardiac muscle tissue development	5.35E-03	10.99601
	Biological process involved in interaction with host	6.16E-03	10.30876
	Striated muscle tissue development	6.16E-03	10.22356
**Cluster 6**	Complement activation	6.63E-05	27.34977
	Granulocyte chemotaxis	6.38E-04	15.52284
	Humoral immune response	3.45E-07	15.21444
	Response to interleukin-1	7.61E-04	14.44894
	Granulocyte migration	1.07E-03	12.90663
	Cartilage development	2.01E-03	10.8367
	Regulation of leukocyte migration	6.97E-04	10.6033
	Regulation of angiogenesis	2.31E-04	10.40885
	Extracellular matrix organization	6.21E-05	10.35439

	Extracellular structure organization	6.21E-05	10.32531

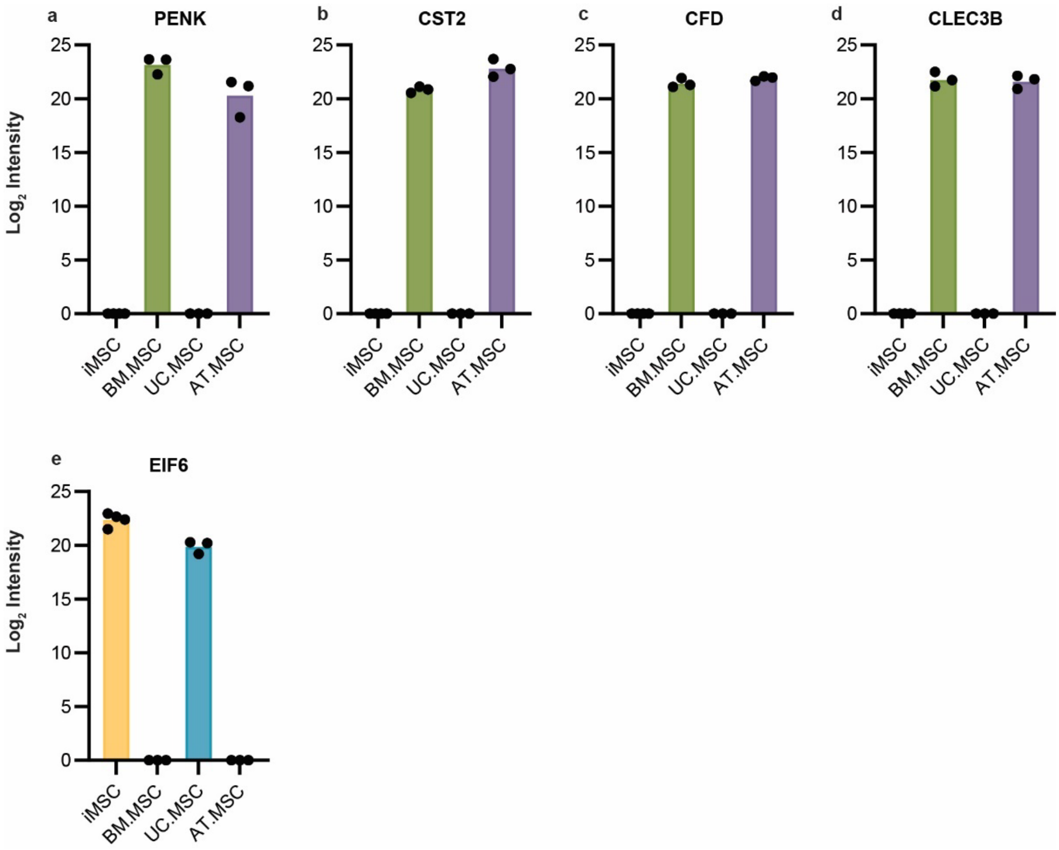

Supplementary Figure 9. Intensity of PC1 loading proteins across licensed MSCs. Bar plots show mean log_2 protein intensity for **(a)** PENK, **(b)** CST2, **(c)** CFD, **(d)** CLEC3B, and **(e)** EIF6 with individual points indicating the intensity of proteins in CM from each MSC line.

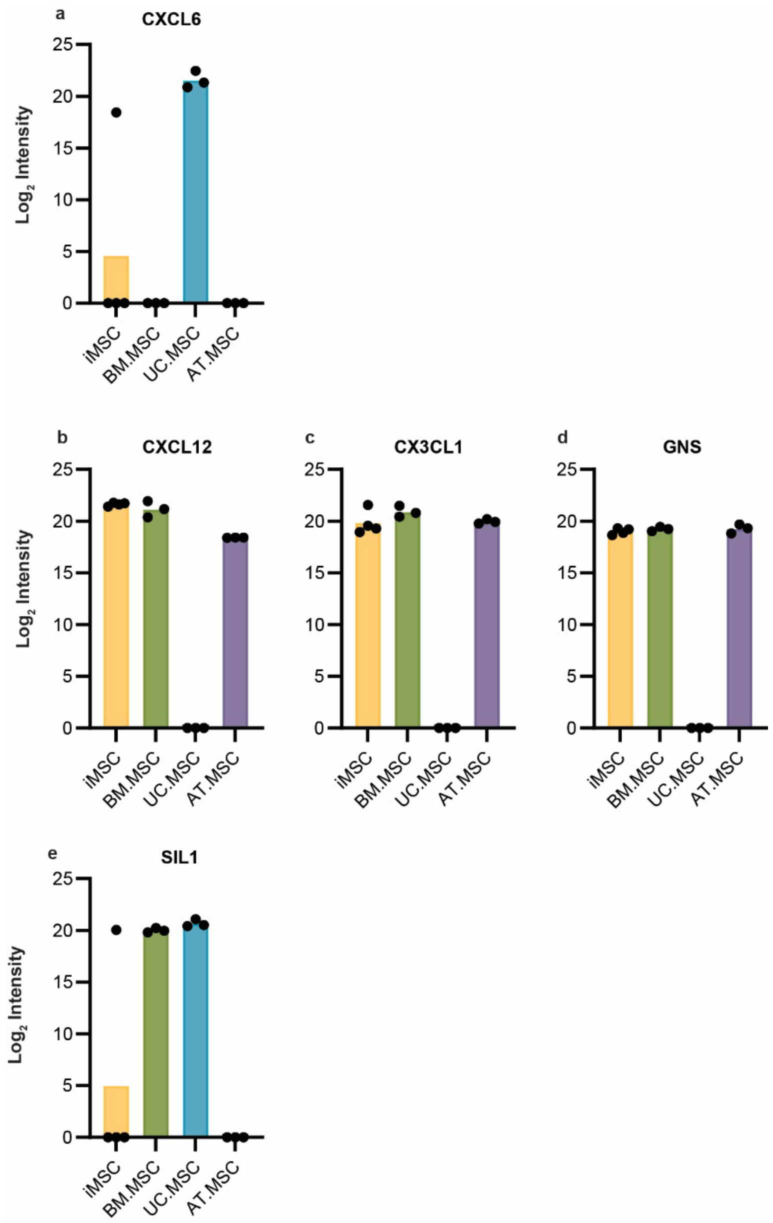

Supplementary Figure 10. Intensity of PC2 loading proteins across resting MSCs. Bar plots show mean $\log_2$ protein intensity for **(a)** CXCL6, **(b)** CXCL12, **(c)** CX3Cl1, **(d)** GNS, and **(e)** SIL1 with individual points indicating the intensity of proteins in CM from each MSC line.

Supplementary Table 4. Enriched processes between licensed MSC secretomes Pathway enrichment performed using iDEP 1.1[96]

	Pathway	FDR	Fold Enrichment
**Cluster 2**	Nucleobase metabolic process	1.50E-03	17.29211
	Nucleoside monophosphate biosynthetic process	2.11E-03	14.14809
	Trabecula morphogenesis	2.93E-03	12.45032
	Regulation of telomere maintenance via telomerase	4.24E-03	10.73304
	Positive regulation of telomere maintenance	2.49E-03	9.70155
	Positive regulation of DNA biosynthetic process	2.55E-03	9.455941
	Regulation of telomere maintenance via telomere lengthening	6.66E-03	9.291286
	Telomere maintenance via telomerase	8.18E-03	8.527618
	RNA-templated DNA biosynthetic process	8.60E-03	8.41238
	Endosome organization	4.87E-03	7.781452
**Cluster 3**	Positive regulation of protein phosphorylation	3.97E-02	6.653718
	Positive regulation of phosphorylation	4.19E-02	6.008095
	Positive regulation of phosphorus metabolic process	4.44E-02	5.476681
	Positive regulation of phosphate metabolic process	4.44E-02	5.476681
	Response to other organism	4.19E-02	4.061027
	Response to external biotic stimulus	4.19E-02	4.053989
	Proteolysis	3.97E-02	4.019672
	Response to biotic stimulus	4.44E-02	3.962425
	Defense response	3.97E-02	3.887069
	Response to external stimulus	3.85E-02	3.425376
**Cluster 4**	Positive regulation of establishment of protein localization to telomere	1.43E-07	46.68871
	Oxaloacetate metabolic process	2.41E-06	43.23029
	Regulation of establishment of protein localization to telomere	2.51E-07	42.44428
	Regulation of protein localization to Cajal body	2.51E-07	42.44428
	Positive regulation of protein localization to Cajal body	2.51E-07	42.44428
	Regulation of establishment of protein localization to chromosome	4.38E-07	38.90726
	Protein localization to nuclear body	4.38E-07	38.90726
	Protein localization to Cajal body	4.38E-07	38.90726
	Positive regulation of protein localization to chromosome telomeric region	6.91E-07	35.91439
	Protein localization to nucleoplasm	1.02E-06	33.34908
**Cluster 5**	Positive regulation of establishment of protein localization to telomere	1.43E-07	46.68871
	Oxaloacetate metabolic process	2.41E-06	43.23029
	Regulation of establishment of protein localization to telomere	2.51E-07	42.44428
	Regulation of protein localization to Cajal body	2.51E-07	42.44428
	Positive regulation of protein localization to Cajal body	2.51E-07	42.44428
	Regulation of establishment of protein localization to chromosome	4.38E-07	38.90726
	Protein localization to nuclear body	4.38E-07	38.90726
	Protein localization to Cajal body	4.38E-07	38.90726
	Positive regulation of protein localization to chromosome telomeric region	6.91E-07	35.91439
	Protein localization to nucleoplasm	1.02E-06	33.34908
**Cluster 6**	ADP metabolic process	1.50E-03	20.30086
	Cytoplasmic translation	9.58E-07	19.91309
	Purine nucleoside diphosphate metabolic process	1.73E-03	17.90167
	Purine ribonucleoside diphosphate metabolic process	1.73E-03	17.90167
	Ribonucleoside diphosphate metabolic process	2.13E-03	16.40986
	Nucleoside diphosphate metabolic process	3.33E-03	14.16679
	Ribonucleoprotein complex assembly	3.36E-03	9.845918
	Ribonucleoprotein complex subunit organization	3.79E-03	9.528308
	Purine-containing compound biosynthetic process	2.80E-02	7.032799
	ATP metabolic process	3.87E-02	6.192401

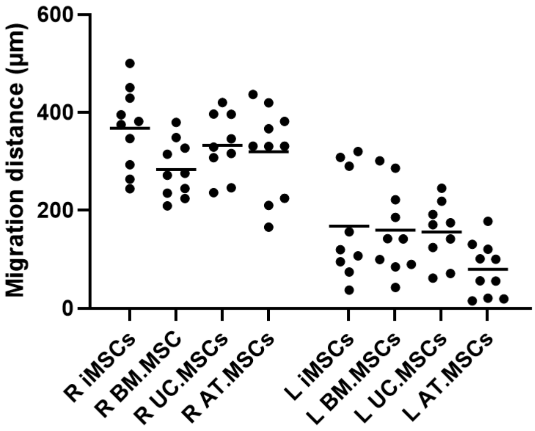

Supplementary Figure 11. Migration distance of hDFs across wound site. Violin plots showing distance of cell migration (µm) of hDFs treated with resting of licensed MSC CM. Migration distance was calculated using live cell imaging over 24 h.

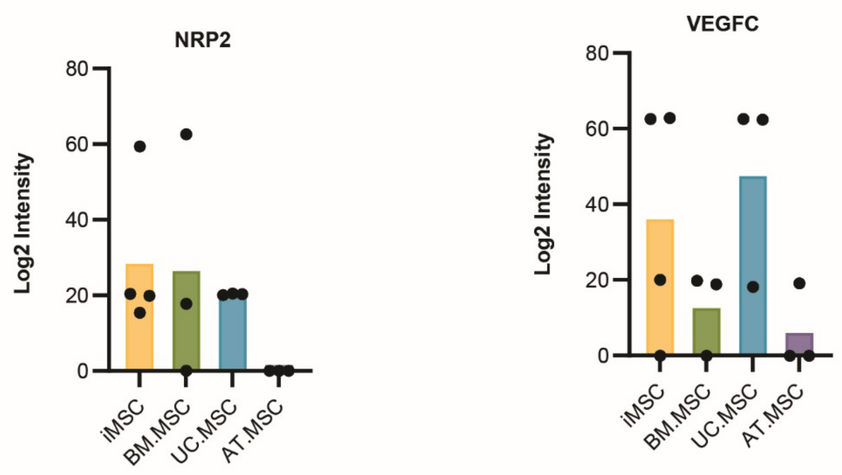

Supplementary Figure 12. Intensity of key angiogenic proteins across resting MSC secretomes. Barplots show mean log_2 protein intensity for **(a)** NRP2, **(b)VEGFC** with individual points indicating the intensity of proteins CM from each MSC line.

Supplementary Data 1 | Processed data matrix including intensity of total and filtered protein lists identified under resting, licensed, and combined parameters. Total protein lists have been filtered to remove contaminants and proteins identified only by sight or reverse sequence. Secreted fragment protein lists have been filtered against GO Term Extracellular Space GO:0005615. LFQ intensity values have been normalised. Where a protein was positively identified in two of three samples within a group the missing value was imputed as the median transformed intensity. Where proteins had at least one positive identification across all samples missing values were imputed from the low end of the Log2 transformed intensity distribution from each individual sample using Perseus (version 1.5.5.3) as suggested by Lazar et al.[95].
